# MTEP: A Matched Temporal Evaluation Protocol for Cognitive Fatigue Detection from In-Task Self-Reports

**DOI:** 10.3390/diagnostics16213556

**Published:** 2026-10-28

**Authors:** Fathe Jeribi, Mousa Mohammed Khubrani

**Affiliations:** 1 Department of Computer Sciences, College of Engineering and Computer Sciences, Jazan University, Jazan 82817, Saudi Arabia; 2 King Salman Center for Disability Research, Riyadh 11614, Saudi Arabia

**Keywords:** cognitive fatigue, evaluation design, report-independence, self-report labels, matched case–control design, validity certificates, EEG, machine learning

## Abstract

**Background/Objectives**: Fatigue models are often trained on labels that participants produce, by an action, while the sensors record. We aimed to certify that an evaluation design’s estimates reflect the state rather than the report. **Methods**: We present MTEP, a matched temporal evaluation protocol delivering label and feature separation, participant-level generalisation, within-recording position balance and report-independence, meaning that no window used to predict a report overlaps the act producing it. We certify report-independence by lead displacement and position balance with linear and nonlinear models, calibrate both certificates with injected effects of known amplitude and shape, compare four evaluation designs on corpora of known truth, and apply MTEP to CogBeacon and CL-Drive. **Results**: On corpora of known truth, MTEP returned the correct verdict in 78 of 80 cases against 58–62 for designs in current use, which concluded that a detector works in 16–19 of 20 corpora containing only the reporting act (MTEP: 1 of 20). The report-independence certificate had a specificity of 39/40 and detected an act-borne component of 0.05 local standard deviations in 30/40 replicates; the nonlinear position certificate detected imbalances the linear test missed (10/10 vs. 0/10). On CogBeacon, MTEP certified at a lead of one round, with pooled ROC-AUC 0.659 carried by task performance. Our model exceeded every published single-classifier accuracy on the same EEG features and protocols, yet a sensor-free clock matched or exceeded published accuracies on both corpora. **Conclusions**: Report-independence is a distinct requirement that existing designs do not imply; it can be checked from the collection protocol and certified on the evaluation sample.

## 1. Introduction

Machine learning is increasingly asked to recognise internal human states such as fatigue, workload, stress, pain and affect. Unlike most applied classification targets, these have no external, independently observed event to serve as ground truth: the label has to come from the person, and in the designs that yield the richest data it comes from them during the recording, as an action performed while the sensors are running [1,2].

That arrangement is what makes such corpora valuable, since a report entered in the moment is time-stamped to the unit and supports event-level targets that an end-of-session questionnaire cannot. It also places a requirement on the evaluation design. The report is something the participant does with the body the sensors are recording: they move a hand to the button, look away from the task, and take longer over the round in which they press. A design that is to estimate how well the sensors track the state must therefore ensure that the window it scores does not overlap the act that produced the label. We call that property report-independence.

Report-independence is one of four properties an evaluation design has to deliver in this setting (Section 2.1). The other three, separation of label and feature construction, participant-level generalisation and position balance within the recording, are well served by designs already in use, and we adopt them. Report-independence is a timing property that none of them implies: they govern which files a pipeline may read, whose data appear where and where a window lies, whereas report-independence governs what a window contains at the instant the label is made. To our knowledge it has not previously been stated as a requirement on an evaluation design, nor decided by a test.

How much it matters is an empirical question, and on the corpus studied here the answer is large. A design that holds the other three properties reaches a pooled ROC-AUC of 0.888 with the reporting round inside the window and 0.659 with it excluded, while the position-only model returns 0.493 in both cases and so raises no objection to either. Under the protocols and metrics of the three published CogBeacon studies, our model also exceeds every published single-classifier accuracy on the same EEG features (Section 3.1), so the protocol is shown on a model that already outperforms published work; the same comparison shows that those accuracies move with the evaluation design, which is the reason the protocol is needed.

The design’s second contribution is that its guarantees are certified rather than asserted: both position balance and report-independence are tested on the evaluation sample itself, and both tests are calibrated against effects of known size and known temporal shape, so that a pass carries a quantity a reader can act on.

We demonstrate the design on the CogBeacon corpus [1], one of the few public datasets recording EEG, facial behaviour, task performance and real-time self-report concurrently, and therefore one on which both requirements bind and can be shown to bind.

### 1.1. Cognitive Fatigue and Its Measurement

Cognitive fatigue is distinguished from sleepiness and from peripheral muscular fatigue and is characterised by a decline in the efficiency of controlled attention under sustained demand [3]. Assessment has relied on subjective instruments such as the NASA Task Load Index [4] and on performance probes such as the psychomotor vigilance task [5], both administered intermittently and both interrupting the activity being measured, which motivated the search for continuous physiological correlates. Electroencephalography has been the dominant one: increased theta and alpha band power and a declining beta-based engagement index recur across laboratory, driving and operational settings [6,7], most recently with reduced electrode montages evaluated under leave-one-participant-out validation [8]. Ocular and facial measures such as blink dynamics and eye closure add a behavioural channel [9], and task telemetry such as response-time variability a third.

### 1.2. Multimodal Benchmarks and Self-Reported Ground Truth

Because no objective physiological definition of cognitive fatigue exists, supervised approaches require an operational label, which in practice is a subjective report. CogBeacon [1] is representative: participants performed variants of the Wisconsin Card Sorting Test [10] while EEG, facial landmarks obtained by regression-tree alignment [11] and per-round task metrics were recorded and pressed a button whenever they had difficulty keeping up.

### 1.3. Evaluation Validity and Where Report-Independence Sits

The methodological literature has documented how strong classification results can arise from evaluation design rather than from signal. Leakage, in which the target becomes inferable through a route that would not exist at deployment, has a developed taxonomy and a documented effect on reported performance across fields [12,13,14,15]. Cross-validation that ignores grouping structure is optimistic [16,17], as measured directly for translational EEG [18], and small samples widen error bars until reported differences stop being interpretable [19]. Paired non-parametric tests [20] and permutation nulls [21] are recommended checks, treated as expected in neighbouring fields [22]. Subject-independent validation is now common in fatigue detection and we adopt it throughout [8,23], though its accuracy falls markedly once the task resembles realistic multitasking [24].

Report-independence sits at a different level from these. Leakage taxonomies are organised around data handling: feature and target boundaries, train and test separation, preprocessing applied before splitting. Report-independence is instead a property of how the label is physically produced; it is consistent with faultless data handling, and it is stated as something a design guarantees and a test certifies, which is what C1 to C3 and the displacement certificate supply. Work on shortcut learning and Clever Hans effects locates an incidental cue by inspecting a trained model [25,26]; here the cue is predictable in advance from the collection protocol, so it can be designed out before training. Reactivity in experience sampling [27,28] and label quality in affective computing [29,30] both ask whether the report is a good measurement of the state; MTEP addresses the question neither literature asks: what the sensors record while the report is being made and which is the quantity the certificate estimates. Designs built on temporal ordering, such as forward-chaining validation, secure a property about information from the future of the window, whereas report-independence concerns information strictly concurrent with the label, so it is orthogonal to them and composes with them.

### 1.4. Model Architectures and Reported Performance

Architecturally, the field has followed the wider trajectory of deep learning on physiological signals, from compact convolutional and deep-belief networks [31,32,33,34] to attention-based, residual and multi-domain fusion designs [35,36,37,38,39]. Reported accuracies in this line of work are often very high, and reviews note the distance between such figures and deployable performance and how rarely they transfer to new participants [38,40].

### 1.5. Contributions

The contributions of this work are as follows.

1. MTEP, a matched temporal evaluation protocol for corpora whose labels are produced by an in-task report, combining label and feature separation, within-recording matched case–control sampling with position balancing, grouped leave-one-participant-out evaluation and report-independent windowing, released as an open implementation.
2. Report-independence as an explicit design property: its definition, the conditions under which it holds automatically and those under which it must be engineered, and a proof that it is necessary for an estimate to be read as state detection rather than as report detection; it is secured by construction, every estimate being read at a lead of at least one unit, and certified on the evaluation sample.
3. Certificates for both guarantees, derived from the structure of the design rather than fitted to any corpus: because the act is confined to the units around the report while a state is autocorrelated across units, displacing the evaluation window separates them, and a design is certified at the smallest lead beyond which every further displacement changes nothing material, which locates preparation for a report as well as the report itself; position balance is certified by five linear and nonlinear position-only models, with a check for interference that persists after the report.
4. Calibration of both certificates against effects of known amplitude and known temporal shape injected into a real feature matrix, reporting specificity, sensitivity, and whether a certificate distinguishes a reporting artefact from a genuine state that produces discrimination of the same size; on corpora of known truth, MTEP returns the correct verdict in 78 of 80 cases against 58 to 62 for designs in current use, and the nonlinear position certificate detects an imbalance in 10 of 10 replicates that the linear test detects in 0 of 10.
5. A requirement table by which a corpus can be checked against the four properties before any modelling, and a complete application to CogBeacon and to a second corpus with a spoken report (CL-Drive), where our model exceeds every published single-classifier accuracy on CogBeacon and the published ten-fold accuracies for two of the three comparable modality sets on CL-Drive, and where a sensor-free clock measures how much of each published accuracy the evaluation design contributes.

Section 2 states the four properties, derives the certificate and describes the corpus, the protocol and the calibration of the certificates. Section 3 first compares our model with published results on CogBeacon under their own protocols, then reports the calibration and the application of MTEP to CogBeacon and CL-Drive, Section 4 discusses the results and their limits, and Section 5 concludes.

## 2. Materials and Methods

### 2.1. The Four Properties

#### 2.1.1. What an Evaluation Design Has to Guarantee

A recording session yields units t = 1, …, T, which may be task rounds, fixed-length epochs or any other unit at which features and labels are aligned. At unit t, a sensing apparatus records a vector x(t). The quantity of interest is a latent state f(t). The available label is not f(t) but a report r(t) in {0, 1}, where r(t) = 1 means the participant performed a reporting act a(t) at unit t. A model is trained on a window X(t) = (x(t − W + 1), …, x(t)) and scored on how well it predicts r(t). For that score to be read as a measure of how well the sensors track f, the design has to deliver four properties.

P1, label and feature separation. The report stream contributes the label and nothing else; no feature is computed from it.

P2, participant-level generalisation. No participant contributes to both the training and the evaluation of the same model, so the score is not inflated by the model recognising individuals.

P3, position balance. Within the evaluation sample, where a window lies inside its recording carries no information about the label, so the score is not inflated by the model reading the clock.

P4, report-independence. No window used to predict a report contains a unit at which a reporting act occurred, so the score is not inflated by the model recognising the act that produced the label.

Table 1 sets out what each property controls, what it leaves open, how the design secures it and how it is verified. The four govern different stages of the evaluation, so a design can hold three and leave the fourth to chance. Grouped and subject-independent cross-validation secures P2, and temporal blocking and matched or covariate-balanced sampling secure P3; each governs which rows are compared with which, and none constrains what a row’s window contains at the moment the label is made. Nor does any of them verify its own guarantee on the sample in hand, which is what the last column adds. P1 and P2 are standard and are secured as described in Section 2.2.4 and Section 2.3.1, the value of grouped cross-validation in repeated-measures data being well established [16,17,41]. The remaining two are the design problem.

#### 2.1.2. Report-Independence

**Definition** **1** (report-independence)**.** *An evaluation is report-independent when no feature window used to predict a report contains a unit at which a reporting act occurred.*

Whether the property holds without being engineered depends on three characteristics of the collection protocol, which can be read off a data statement. Report-independence is automatic unless all three hold.

C1, the label is act-borne. It is produced by an action of the participant, rather than by an external observer, an offline annotation, or an instrument independent of the participant’s body.

C2, the windowing overlaps the act. The window from which features are computed includes the unit or units during which the act is performed.

C3, the sensors respond to the act. At least one recorded channel changes with the act, through movement, gaze, posture, vocalisation, or the timing of a concurrent task.

**Proposition** **1.** *If C1, C2 and C3 hold, then an evaluation that is not report-independent does not identify state detection: there exists a classifier on X(t) whose discrimination exceeds chance even when f(t) is statistically independent of r(t).*

**Proof of** **Proposition 1.** Write the recorded vector as x(t) = g(f(t)) + h(a(t)) + e(t), separating the signature of the state in the sensors, the component attributable to the act with h(0) = 0, and everything else. By C1, r(t) = 1 if and only if a(t) is non-zero; by C2, x(t) enters X(t). The difference in expectation of X(t) between the two label values is therefore h(a) plus the difference contributed by g(f(t)), and when f is independent of r the second term vanishes, leaving h(a), which by C3 is non-zero in at least one coordinate. A likelihood-ratio rule based on that difference attains ROC-AUC above one half. □

The argument asks for nothing beyond the three conditions. It makes report-independence a necessary condition that can be checked from the collection protocol before any model is fitted, and it shows how little is required for it to bind: no relationship whatever between the sensing streams and the state of interest. A corpus in which EEG carries nothing about fatigue will still support above-chance fatigue classification if the window contains the press. How large the resulting advantage is depends on the separability of h(a) from the distribution of e, and Section 3.2 measures how small h can be while the certificate still detects it.

None of the other three properties implies report-independence, because each is about something else. P2 concerns whose data appear where, whereas the act-borne component is a within-session effect present identically in training and held-out participants, so holding out participants leaves it intact. It is also more consistent across people than the state signal, because the biomechanics of pressing a button vary less between individuals than the physiological expression of fatigue, so a design that lacks P4 can generalise to unseen participants unusually well.

P3 concerns where a window lies, and a design can be exactly balanced in position while placing the reporting unit inside every case window. Ours was: on CogBeacon, the position-only model returned a pooled ROC-AUC of 0.493 both when the case window ended on the press round and when it ended one round earlier, while the estimates from those two designs differed by 0.228.

P1 concerns provenance. Asserting that no feature is computed from the report stream is worth doing, but the act-borne component reaches the model through the sensor streams, which the feature pipeline is entitled to read, so P1 holds while P4 fails. Artefact correction [42,43] sits alongside rather than in place of P4: the act-borne component is synchronous with the label, so an estimate obtained after correction is valid only to the extent that correction was complete, which cannot be verified from within the study, and the same act changes the facial video, the head pose and the duration of the round, streams for which no comparable correction stage exists.

#### 2.1.3. Certifying Report-Independence by Lead Displacement

The two components differ in their temporal support, and that difference makes a certificate possible. The term h(a(t)) is confined to the units at which the act occurs and is, by construction, a discontinuity; the term g(f(t)) is the signature of a state that accumulates over a session and is therefore autocorrelated across neighbouring units. Filtering cannot separate them, because they overlap in frequency and are aligned in time. Displacement can.

Let the case window end L units before the reported unit and evaluate the same design at successive values of L under identical folds, features and models. Where the window contains the act, discrimination falls sharply between L = 0 and L = 1 and changes comparatively little thereafter; where the sensors genuinely anticipate the state, it declines gradually, at the rate at which f decorrelates, with no step at any particular lead. The discontinuity, not the level, is the diagnostic quantity.

The statistic is therefore D(L) = AUC(L − 1) − AUC(L), the difference in pooled ROC-AUC between two adjacent designs computed on the same participants, folds, estimator and hyperparameters, with a 95% interval obtained by resampling participants with replacement and recomputing both terms. The certified lead L* is the smallest lead beyond which every further displacement step in the sweep is immaterial: displacing the window further changes nothing, so the estimate has stabilised on the state signal the design set out to measure. The interval rule asks whether the interval for D excludes zero, and so whether any discontinuity is present; the magnitude rule asks whether D reaches a materiality threshold, for which we adopt 0.10, justified from the calibration in Section 3.2 rather than by convention, and so whether the discontinuity is large enough to change what the estimate means.

Because the certificate keys on the step rather than on the level, it is insensitive to how much genuine signal surrounds the report; Section 3.2 measures this directly against injected components of both shapes. A certificate that is not granted at L = 0 is then a statement about where the estimate should be read, not about the corpus: the analysis belongs at a lead of at least one unit, and what remains there may be smaller than the figure available at L = 0 but, unlike it, is interpretable.

Definition 1 and the certificate establish different things, and MTEP uses both. Definition 1 is secured by construction: when C1 to C3 hold, every MTEP estimate is read at a lead of at least one unit, so no window used to predict a report contains the reporting unit, whatever the certificate returns. The certificate establishes what construction cannot: that the act has no material influence on the estimate through the units adjacent to the reporting unit, where preparation for a press begins and its consequences may persist. It is a measurement of influence at a calibrated sensitivity (Section 3.2), and the estimate is reported at L_rep = max(1, L*). A certified lead of zero, which EEG returns on CogBeacon (Section 3.5), therefore measures that the act leaves no material trace in that stream, and the EEG estimate, like every other, is read at L = 1, the smallest lead Definition 1 allows.

#### 2.1.4. Where the Requirement Binds

Because C1 to C3 are stated in terms of how the label is produced and how the features are windowed, a corpus can be checked against the requirement from a data statement, before any model is fitted. Table 2 does this for the label-production mechanisms common in human state recognition.

Three observations follow. First, the requirement attaches to the protocol by which the label was collected, not to the modality, the model or the sample size. Second, it binds in the largest category in practice, in-task reporting, for the same reason those corpora are valuable: a report entered in the moment is entered while the sensors are running. Third, a questionnaire administered at a block or session boundary meets the requirement or not depending entirely on whether features are permitted to run up to the moment it is answered, a decision rarely stated in reports and one that costs nothing to state.

The seventh row fails C1, so report-independence holds automatically, but it raises a distinct question: when the label is an instrumented property of the participant’s own behaviour, such as a lapse in a vigilance task, the label and a feature may be the same measurement. That concerns P1 and P3 rather than P4.

### 2.2. Corpus, Labels and Features

#### 2.2.1. Dataset

All analyses use the publicly released CogBeacon corpus [1]: four-channel Muse EEG (raw signal at 220 Hz with absolute and relative band powers at 10 Hz), 68 facial landmarks and a face bounding box at 2 frames per second, per-round task-performance logs, and a real-time self-report recorded as the cumulative number of button presses at each round.

CogBeacon is one of the few corpora on which report-independence can be observed at all. The label is produced by an action taken during the task, so the requirement binds; the time of that action is recorded at the resolution of the analysis unit, so a lead can be applied and the certificate computed; and several streams are recorded concurrently, so a trace of the act can be separated from a trace of the state (Section 3.5).

The release as distributed contains 77 session folders from 20 participant identifiers: 19 identifiers with four sessions each, which are the 76 sessions from 19 users that the documentation describes, and one further identifier with a single session in which the button was never pressed. We processed the release as distributed, and 6972 rounds carried complete EEG and task-performance records and entered the analysis. Having no press, the additional session contributes neither a case nor a control to the matched design, so every MTEP estimate, certificate and calibration is identical with or without it; it enters only Study 1, the reproductions of published protocols and the full-stream evaluation, as negative rounds. CogBeacon contains no Karolinska Sleepiness Scale or other graded subjective fatigue scale; the only subjective signal is the press count.

#### 2.2.2. Label Construction

The cumulative press count admits two binary targets, and the distinction proves consequential.

The state label marks a round positive if the participant had pressed the button at least once at or before that round. This is the labelling implied by treating self-report as a running indicator of fatigue status, and it yields a moderately balanced problem; Section 3.4 shows that position balance cannot be established for it within a session.

The event label marks a round positive if a new press was registered at that round, that is, if the cumulative count increased. Extending the target to a horizon of D rounds gives event (D), positive when at least one new press falls in the interval from the current round to D rounds ahead.

Across the corpus there were 79 press events in total, or 1.13% of rounds, 42 of the 77 sessions contained no press at all, and the presses came from 15 of the 20 participant identifiers.

The press is a self-report. Participants differ in how they perceive difficulty and in the threshold at which they act on it, so the event label is a noisy, participant-dependent measurement of any underlying state: a report may lag or lead the state, a state may pass unreported, and a press may be made without one. We therefore read the target as what it operationally is, an upcoming self-report of difficulty keeping up, which is the label CogBeacon provides for cognitive fatigue, and we use state detection in the sense of Section 2.1.1: discrimination that comes neither from the act of reporting nor from the clock. Section 3.2 tests MTEP’s verdicts under each of the three forms of label noise.

#### 2.2.3. Feature Extraction

Each round was summarised by 97 values in three groups (full inventory with missingness in Table S4). The 72 EEG features are, for each of the four electrodes (TP9, AF7, AF8 and TP10), the mean of all five absolute and all five relative band powers, the standard deviation of the relative bands, the theta/alpha ratio, the beta/(theta + alpha) engagement index and the mean signal-quality indicator.

The 13 facial features are the height-to-width ratio of the eye and mouth landmark extents, as mean and standard deviation over the round’s frames (a simpler measure than the eye aspect ratio used in blink detection); the width, height and position of the face bounding box; and the frame-to-frame displacement of the landmark centroid normalised by box width, as mean, standard deviation and maximum, with the standard deviation of the normalised centroid position.

The 12 task and timing features are difficulty level, round score, response time, correctness, question index, perseveration flag, normalised round position, the within-round increments in perseverative and non-perseverative errors, a response-time deviation relative to the preceding ten rounds, a ten-round running accuracy, and the number of video frames in the round, which measures round duration at 2 frames per second and is therefore grouped with task timing. Normalised round position is a diagnostic variable, not a feature available at deployment.

Relative band features of the third channel were missing for 32.8% of rounds and the facial extent ratios for 11.2%; missing values were imputed with the training-fold median, and all normalisation statistics were fitted on training folds alone.

#### 2.2.4. Keeping Label Construction Separate from Feature Construction

Because the self-report stream is both the only available label and a plausible input, the two roles are separated in code. Labels are produced by a function that reads only the fatigue_self_report directory and features by functions that read only the eeg, face_keypoints and user_performance directories, with no shared state, and a closing check fails the build if any feature name contains a label-related term. This guards against a self-report column entering the feature set; it cannot detect traces of the reporting act in the other streams, which is the role of the lead (Section 2.3.3).

### 2.3. Study Designs

#### 2.3.1. Study 1: Conventional Protocol

Study 1 applies the protocol typical of this literature: sequences of 10 consecutive rounds within each session at stride 1, labelled by the final round, evaluated by grouped leave-one-participant-out cross-validation in which every session of a held-out participant forms the test fold. Within each training set one eighth of the training participants (at least one) were held out from all models and used only for early stopping of the Transformer.

Fold-wise ROC-AUC exists only for the 15 participants whose held-out windows contain both classes; the others never pressed and enter only the pooled out-of-fold estimate.

#### 2.3.2. Establishing Position Balance

We added a clock baseline to Study 1: a logistic regression given only the normalised position of the round within its session. Because classical models receive the mean, standard deviation and final value of each feature across the window, the baseline sees three summaries of this single variable. A second clock uses the number of rounds elapsed since the session began, which is available at deployment. If a model with no access to physiology, behaviour or task performance performs competitively, P3 does not hold for the target as defined.

#### 2.3.3. Study 2: Within-Session Case–Control Design

Section 3.4 shows that the state label cannot be decorrelated from session position by matching, because within a session position determines the label exactly. We therefore adopted a case–control design on fatigue events, matched inside the session.

A case is a window of W rounds that ends L rounds before a round in which a new press occurred. The lead L is what makes the design report-independent: with L ≥ 1 the round in which the button was pressed, and with it any movement, gaze shift or delay caused by pressing, lies outside the case window. Case windows that contain any press round, which happens when presses cluster, were discarded. Controls are windows from the same session whose end lies at least g and at most m rounds from every press and whose span contains no press round. For each case, k controls were selected so that they fall on both sides of the case where the session allows, by steering the running mean offset towards zero, with each pick drawn at random from the three nearest remaining candidates on the chosen side; no control window is used twice. A case and its controls form a matched set, and matched sets are disjoint.

The default design uses W = 10, g = 5, m = 30, k = 3 and L = 1. It yielded 68 cases and 204 controls from 34 sessions and 15 participants; of the 79 presses, 2 fell within the first W rounds of a session and 9 were discarded because the case window contained an earlier press. The mean absolute offset between a control and its case was 9.3 rounds, and 34% of controls lie after their case. Case and control windows from the same set may overlap in time: 61% of controls overlap their case window, sharing 4.5 rounds on average. No window is used twice, matched sets share no window, and because every window of a participant falls in the same fold, overlap never places a round on both sides of a training and evaluation split. Section 3.6 reports variants that draw controls only before or only after their case, and one in which no two windows of a set share a round.

Because the design has free parameters whose choice could in principle drive the result, we repeated the analysis at L = 1 across 33 configurations: window lengths of 5, 10 and 15 rounds, minimum gaps of 3, 5 and 10 rounds, and maximum offsets of 20, 30 and 50 rounds with k = 3 (27 configurations), plus k = 1, 2 and 5 and three further sampling seeds at the default configuration, recomputing the position-only baseline in each. Because the certificate is itself a difference of pooled ROC-AUCs computed at a fixed window length, we separately repeated the displacement sweep on all features with each of the three classical estimators and with the random forest at window lengths of 5, 10 and 15 rounds.

#### 2.3.4. The MTEP Protocol

**Figure 1:**
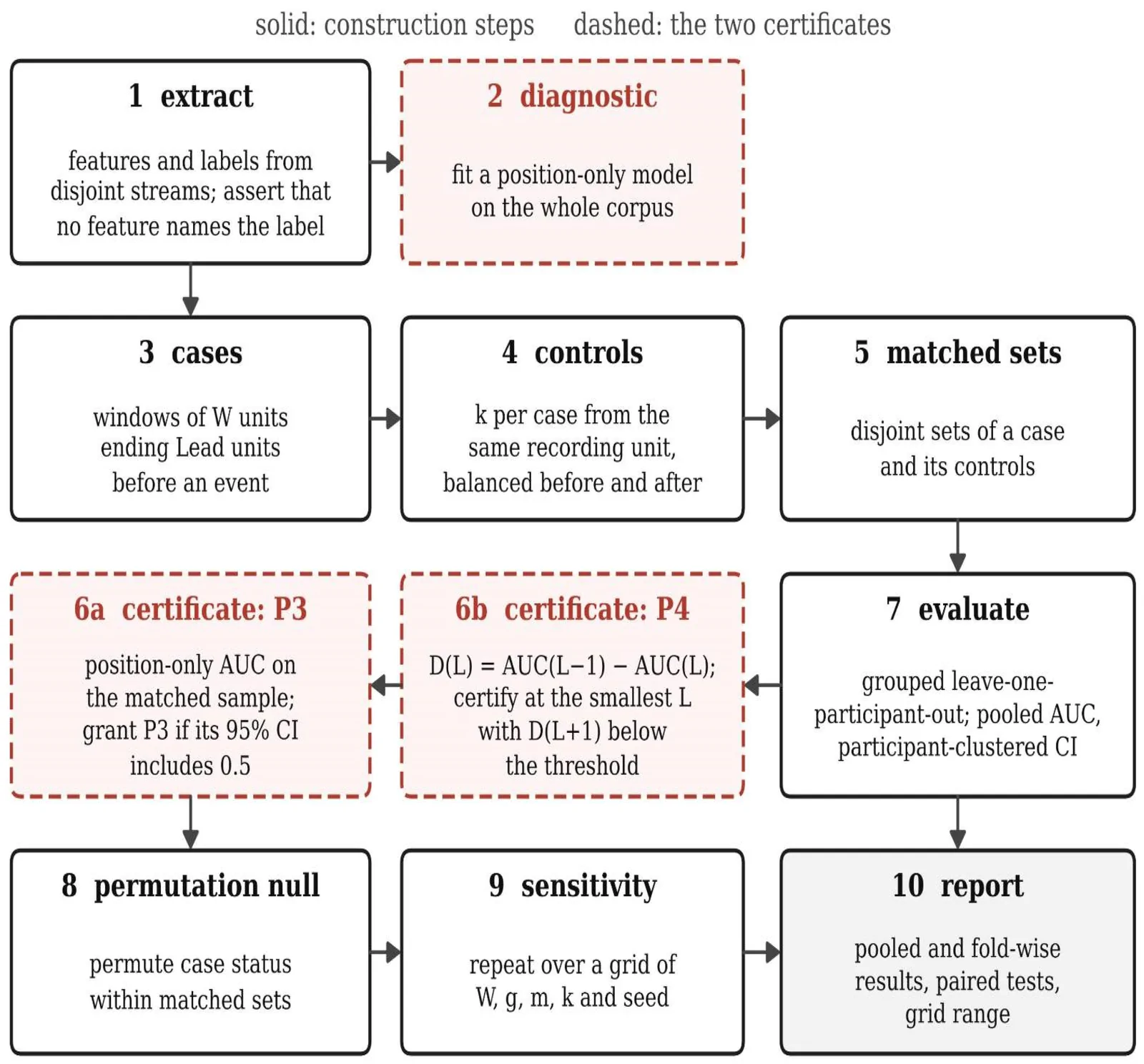
The MTEP design. Solid boxes: construction steps. Dashed boxes: the two certificates. Step numbers are those of Algorithm 1.

Algorithm 1 states the protocol and Figure 1 shows it as a flow diagram, for a corpus organised into recording units nested within participants with an event-valued label stream and one or more feature streams. Steps 6a and 6b make the guarantees checkable rather than asserted: 6a establishes P3 on the evaluation sample and 6b locates the lead at which P4 holds. Each certifies the property it names on the evaluation sample itself, so a reported estimate carries both guarantees with a measured specificity and sensitivity.
**Algorithm 1.** MTEP: Matched Temporal Evaluation ProtocolInput :   corpus C of sessions s, each with participant id u(s),  event stream L(s), feature streams F(s)  window W, lead Lead >= 1, gap g, max offset m, controls kOutput:   performance estimate, or FAIL   1  X <- extract(F(s)) for all s           // reads F only  Y <- events(L(s))  for all s           // reads L only  assert  name(x) references no element of L   for all x in X   2  diagnostic: fit position-only model on (pos, Y) under grouped CV  if AUC_diag >> 0.5 then P3 does not hold for this target   3  cases   <- windows of length W ending Lead units before an event,         containing no event unit   4  controls <- for each case c in session s: k windows from s with         g <= |distance to every event| <= m, containing no         event unit, balanced before/after c, each used once   5  M <- disjoint matched sets {case + its controls} 6a  CERTIFY (position balance): refit position-only models on M (linear, spline, forest, boosting, nearest-neighbour) grant P3 if the 95% CI of every pooled AUC_diag(M) includes 0.5 6b  CERTIFY (report-independence): repeat steps 3-7 with Lead = 0,1,2,3 D(L) <- AUC(Lead = L - 1) - AUC(Lead = L), participant-clustered CI L* <- smallest L with D(M) < delta* for every M > L;  report the estimate at max(1, L*)   7  evaluate models on M by grouped leave-one-participant-out CV;  pooled AUC with participant-clustered bootstrap CI   8  permutation null: permute case status within matched sets, repeat 7   9  sensitivity: repeat 3-8 over a grid of (W, g, m, k, seed) 10  report pooled and fold-wise results, paired tests with  wins/ties/losses, the grid range, and the AUC against earlier controls alone (persistence check)

Steps 1 to 6b are the protocol and steps 7 to 10 the reporting standard we apply to it. The released implementation exposes each step separately, so that a corpus with a different structure, for example one whose recording unit is a working shift, can substitute its own feature extractor and event definition while retaining the diagnostic, the matching and both certificates.

#### 2.3.5. Evaluation on the Full Recording Stream

The matched design measures discrimination between report-preceding and report-distant windows at a prevalence of 25%. To measure what the certified detector does when it scores a whole recording, each model was trained exactly as in Study 2, on the matched sets of the other participants at L = 1, and applied to every window of W rounds in the held-out participant’s sessions; a window is positive when a new press occurs in the following round. Participants who never pressed are scored as well, so every one of their windows is a potential false alarm. We report pooled ROC-AUC with a participant-clustered bootstrap interval, PR-AUC against the stream prevalence, and, at alert budgets of 5% and 10% of windows, the precision, its lift over prevalence, and the share of presses flagged one round ahead or within three rounds ahead.

### 2.4. Models and Statistical Analysis

#### 2.4.1. Models

We compared four classifiers under identical folds, features and normalisation, without hyperparameter tuning for any model (settings in Table S6), and in every outer fold all models were trained on exactly the same participants. The Transformer encoder [46] used a linear input projection to 48 dimensions, sinusoidal positional encoding, two encoder layers with four attention heads, and a linear head on the final time step, trained with class-weighted binary cross-entropy and early stopping on validation ROC-AUC. Logistic regression, a random forest [47] and, in Study 1, a support vector machine were fitted on window summaries formed from the per-feature mean, standard deviation and final value across the window, with balanced class weights.

#### 2.4.2. Statistical Analysis

The primary summary is the pooled ROC-AUC computed from the concatenated out-of-fold predictions of all held-out participants, with a 95% confidence interval from a participant-clustered bootstrap (2000 resamples of participants with replacement). Fold-wise means and standard deviations are secondary, because in the controlled design several participants contribute only one or two cases, so their fold-wise AUC can take only a few discrete values. Precision–recall summaries are reported alongside ROC-AUC [48].

Comparisons between configurations are paired by participant and tested with the Wilcoxon signed-rank test [20,49] on fold-wise ROC-AUC, discarding zero differences and using the exact null distribution when no zero or tied differences occur and the normal approximation otherwise; we report wins, ties and losses, the matched-pairs rank-biserial correlation, and Holm–Bonferroni corrected *p*-values across the family of 10 comparisons. Significance against chance was assessed by a permutation null [21] that permutes case status within each matched set and repeats the full cross-validation 200 times with the random-forest model whose result is reported. Every value in Section 3 is generated programmatically from the released feature matrix and the stored prediction records.

#### 2.4.3. Reproduction of Published Protocols

To compare with published results on the same data, protocol and metrics, we reproduced the evaluation protocol of each study that reports fatigue classification on CogBeacon, with the EEG features and the cumulative label those studies use, in which a round is labelled fatigued once the participant has pressed at least once in the session. Under the protocol of the dataset paper [1], each of ten folds holds out 20% of the sessions at random, training classes are balanced by down-sampling, and accuracy and macro F1 are averaged over folds. Under that of Ekim et al. [50], rounds are split at random into two halves, repeated 5 times here against 100 in the original; under that of Das et al. [51], rounds are divided by stratified ten-fold cross-validation at the natural class ratio, run once here against ten repetitions in the original. Our model is the random forest of Section 2.4.1, without tuning, applied to the MTEP window summary of the EEG features: the mean, SD and final value of each feature over the ten rounds ending at the scored round. A clock, the same forest given only the round’s position within its session and no sensor, is scored under every protocol as a reference.

### 2.5. Calibrating the Two Certificates

A certificate applied once to one corpus returns pass or fail and nothing else, so we calibrate both certificates against departures of known size and known temporal shape, on the same corpus, estimator and folds as the analyses themselves.

The reference condition has to be a design that satisfies report-independence while otherwise being the real thing. Editing the press rounds out of the feature matrix would introduce a discontinuity of its own at exactly the position the certificate examines, so we leave the feature matrix untouched and move the events. In each replicate the matched design is rebuilt around synthetic events drawn at random within each session, as many as that session has real presses, each at least W + g = 15 rounds from every real press and from every other synthetic event, with the real press rounds blocked so that no case or control window can contain one. The feature covariance, the session and participant structure and the within-session drift are therefore the corpus’s own, while the design is report-independent by construction. Across 20 replicates the designs averaged 66.1 cases and 197.6 controls, close to the 68 and 204 of the real design.

Onto that reference we add a component of known amplitude at the synthetic events. Each event draws a latent magnitude z = a(1 + e), with e standard normal, so that the component has mean amplitude a and varies between occurrences as a real act would, loading with random sign on ten features drawn at random per replicate. Amplitude is expressed in local standard deviations, the median standard deviation of the feature within a window of W rounds, because that is the variability a component has to stand out against once fold-wise scaling has removed between-participant offsets. Feature eligibility, resolution snapping and the check that no injected value can reach an L = 1 window are given in Supplementary Section S10.

Two temporal shapes are injected, and the contrast between them is the point of the exercise. A spike places the component on the event round alone, which is what a reporting act produces and what report-independence excludes. A state spreads it over surrounding rounds with Gaussian weight of width 6 rounds centred on the event, which is what a genuine accumulating state looks like: present before the report, largest at it, decaying after it, and with no discontinuity anywhere. The two differ in shape, not in how much signal they place near the report, so a certificate that claims to key on shape can be held to it. Spike amplitudes are 0, 0.005, 0.01, 0.02, 0.05 and 0.1 local standard deviations and state amplitudes 0.25, 0.5 and 1.0, the latter larger because a component spread over about eleven effective rounds must be larger to produce comparable discrimination.

An extended calibration departs from both shapes in the directions in which a real act and a real report can depart from them, with new seeds and the same construction (Supplementary Section S17). Three act-borne conditions test the assumption that the act is confined to the reporting unit: preparation on the preceding round, with or without the act itself, and heavy-tailed occurrence-to-occurrence magnitudes (Student t, 3 degrees of freedom). Two test persistent reporting interference: a shift that begins at the report and lasts for the rest of the session, at 0.1 and 1.0 local standard deviations, and one that decays with a time constant of five rounds. Four genuine states have non-Gaussian temporal profiles: an abrupt onset three rounds before the report that is then sustained, a linear ramp over the ten rounds before it, an exponential rise with a fast decay after it, and a Gaussian profile with heavy-tailed magnitudes. Three conditions add label noise to a genuine state: the report displaced from the state peak by up to three rounds either way, as many unreported state episodes as reported ones, and half of the reports made without any state. Each condition is evaluated at leads 0, 1 and 2, and 3 for the preparation conditions, and MTEP’s verdict is read at the reported lead.

The position certificate reads only the normalised position of a window, so it is calibrated by varying the design rather than the features. With probability γ, each control is taken from the nearest remaining candidate before its case instead of by the balancing rule, so that γ = 0 is the design as specified and γ = 1 always takes the nearest earlier window. The real design at L = 1 is rebuilt at γ = 0, 0.25, 0.5, 0.75 and 1 over 40 replicates each. Because that imbalance is linear in position, a second sweep introduces one that is not: with probability γ_far, each control is the farthest remaining eligible window on the side chosen by the balancing rule, so controls stay balanced before and after their case but spread away from it. The label then depends on position through the dispersion of controls around their case rather than through their mean offset, which a linear model cannot represent. Each design is scored by the linear position model and by a cubic spline, a random forest, gradient boosting and 25-nearest neighbours on the same position summary, and by a random forest and gradient boosting on a position family that adds question index, elapsed rounds and session length; the revised certificate is granted only when the intervals of all five models on the position variable include 0.5, and the position family is reported alongside as a further check where such variables exist. The sweep uses γ_far = 0, 0.25, 0.5, 0.75 and 1 with 10 replicates each.

Specificity is the proportion of replicates in which a sound design is certified, and sensitivity the proportion in which a design carrying an injected departure is refused; both are reported as counts with Clopper-Pearson 95% intervals. Every setting in the calibration is the one used for the analyses of the corpus itself, and the calibration code reproduces the pooled ROC-AUC of the real design exactly when run without injection.

## 3. Results

### 3.1. Comparison with Published Results on CogBeacon

Three studies report fatigue classification on CogBeacon, each from EEG, under their own protocols and on the cumulative label [1,50,51]. Table 3 scores our model on the same EEG features, under each of these protocols and on the metrics each study reports (Section 2.4.3).

Published values are those reported in the original studies; Das et al. [51] report 93.83% after class balancing by synthetic oversampling, and the natural-ratio figure is the like-for-like one. Ours: untuned random forest on the MTEP window summary of the EEG features (Section 2.4.3). Clock: the same forest given only the round’s position within its session. With all sensor features (EEG, facial and task), the forest reached 0.674/0.584, 98.1% and 99.3%/0.988 under the three protocols. Under the two random splits, the rounds that share a scored round’s window fall on both sides of the split; on single rounds, the forest reached 82.0% and 83.9% with EEG features and 87.4% and 90.0% with all sensor features, and under the session split 0.617 with EEG features. Repetitions: Five random splits and one ten-fold cross-validation, with accuracies varying by SDs of 0.6 and 0.5 percentage points.

On the same EEG features and protocols, our model exceeds every published single-classifier accuracy. Under the random split of Ekim et al., it reached 96.5% against 83.34%, and under the ten-fold cross-validation of Das et al., 98.5% with an F1 of 0.976 against 90.96% and 0.86. Under the session split of the dataset paper, it reached an accuracy of 0.668, above the 0.64 of the best published single classifier and level with the 0.67 of the published ensemble of five classifiers, without any tuning.

The last column of Table 3 is a clock: a single variable giving the round’s position within its session, with no sensor at all. Under the dataset paper’s own protocol, it reached an accuracy of 0.686 and a macro F1 of 0.637, above the published ensemble on both metrics. Under the cumulative label, a round is more likely to be labelled fatigued simply because it comes later in the session, so an accuracy obtained under these protocols, the published figures and ours alike, measures position within the session together with fatigue. The comparison therefore establishes that our model outperforms published work on its own terms, and it shows why those terms need the further guarantees MTEP supplies.

Table 3 also separates two routes by which a protocol shapes accuracy. Under the session split, no round crosses the split, and the ten-round window raised EEG accuracy from 0.617 on single rounds to 0.668, above the best published single classifier. Under the two random splits, single-round EEG reached 82.0% and 83.9%, level with the 83.14% and 83.34% of Ekim et al. and below the 90.96% of Das et al., and the window summary raised them to 96.5% and 98.5%. A window summarises the ten rounds ending at the scored round, and under a random split of rounds the windows of neighbouring rounds, which share most of their rounds with it, fall on both sides of the split, so these windowed figures, like the published random-split figures, measure performance on rounds whose neighbours were seen in training. The comparison Table 3 supports is therefore the one it was built for: on this corpus, accuracy follows the evaluation design, through position under every protocol and through shared rounds under random splits, and that is what MTEP is designed to control.

Table 4 sets MTEP against its baselines at two levels. Panel A compares evaluation designs on simulated corpora of known truth, adding one property at a time to the designs in current use (Section 3.3): MTEP alone withholds the verdict when only the reporting act is present, and it returns the correct verdict in 78 of 80 cases against 58 to 62 for the baseline designs. Panel B compares models under MTEP on CogBeacon, each reported with the guarantees the protocol adds to a single accuracy: an interval, a certified lead, a permutation test and performance on the full recording stream. The multimodal forest reached a pooled ROC-AUC of 0.659 at the certified lead, above logistic regression (0.602), the Transformer (0.610) and every single modality except task telemetry (0.707), while the clock stayed at chance (0.493).

In panel B, matched values are pooled over held-out participants at L = 1 with participant-clustered bootstrap intervals (Section 3.6); the reported lead is max(1, L*), with L* the smallest lead beyond which every further displacement changes the estimate by less than 0.10 (Section 3.5; for LR, Table S9); full-stream ROC-AUC scores every window of every held-out session (Table S9); *p* (perm.) is from 200 label permutations within matched sets (Section 3.8); and — indicates not computed. In panel A, each design adds one property to the one above it and returns one verdict, whether the 95% interval for its pooled ROC-AUC excludes 0.5. Worlds are built on synthetic events so that the truth is known: an act-shaped component of 0.1 local standard deviations carries no state, a smooth state of 1 does. Cells give the number of replicates in which the design concluded that a detector works, with the mean pooled ROC-AUC it reported in brackets; a design is correct when it says yes exactly in the two worlds where a state is present. The random-fold design resamples rows for its interval, as an analyst using random folds would; the other three resample participants.

### 3.2. What a Certificate Is Worth

Table 5 reports the report-independence certificate. On the reference design, which satisfies the property by construction, the displacement statistic averaged +0.017 (SD 0.042) and the design was certified in 19 of 20 replicates under the magnitude rule and 18 of 20 under the interval rule, specificities of 95% (95% CI 75–100%) and 90% (95% CI 68–99%). Sensitivity to a component confined to the reporting round rose with its amplitude: the magnitude rule refused 9 of 20 designs at 0.005 local standard deviations, 17 of 20 at 0.05 and 18 of 20 at 0.1, as the statistic rose from +0.113 to +0.214. The certificate therefore sees departures far smaller than the one the corpus turns out to contain.

The design is rebuilt around synthetic events (Section 2.5), so it is report-independent by construction, and a component of the stated amplitude in local standard deviations is injected on the event round alone (act) or with a Gaussian weight of width 6 rounds around it (state). D is the difference in pooled ROC-AUC between leads of 0 and 1 rounds, as mean (SD) across replicates. A design is certified under the interval rule when the participant-clustered bootstrap 95% interval for D includes zero, and under the magnitude rule when D is below 0.10.

The rows that decide whether the certificate is usable are the last three. A genuine state of amplitude 1.0 raised pooled ROC-AUC at a lead of one round from 0.521 to 0.714, a large and entirely real effect of the kind the design exists to pass through; the displacement statistic nevertheless stayed at +0.019 (SD 0.035), and the magnitude rule certified 20 of 20 such designs, as it did at amplitudes 0.5 and 0.25. The interval rule certified 15 of 20 at amplitude 1.0, reflecting a small genuine step at the peak of the injected bump; that is the price of its greater sensitivity, and the reason we take the magnitude rule as primary and the interval rule as a screen.

The certificate therefore responds to a discontinuity at the report and not to the quantity of signal near it, as Section 2.1.3 claimed: a component that leaves discrimination at 0.521 one round away drives the statistic to +0.188, while one that lifts it to 0.714 leaves it at +0.019. Across all 80 report-independent cells, the 20 reference and 60 genuine-state cells, the largest statistic observed was +0.118.

Those 80 cells also fix the materiality threshold. At 0.10, two of the 80 sound designs are refused (2.5%), while the threshold is reached in 17 of 20 designs carrying a component of 0.05 and in 18 of 20 at 0.1. Lowering it to 0.05 would refuse 19 of the 80 sound designs (24%) in exchange for one additional detection; raising it to 0.15 would refuse none but miss four detections at 0.05. A threshold of 0.10 is where that exchange stops being favourable, and the calibration by corpus size shows it is conservative at every size tested. In an independent second set of 20 replicates at the same size (Table S10), the largest statistic over the 60 sound designs was +0.076, so the threshold refused none of them.

That second set, drawn with new seeds as the full-size arm of the calibration by corpus size, certified 20 of 20 reference designs and refused 13 of 20 and 15 of 20 designs carrying an act-shaped component of 0.05 and 0.1. Pooled over both sets, the certificate certified 39 of 40 reference designs, a specificity of 98% (95% CI 87–100%), refused 30 of 40 designs at an amplitude of 0.05 (75%) and 33 of 40 at 0.1 (83%), and certified all 80 designs carrying a genuine state of amplitude 0.5 or 1.0.

Table S10 repeats the calibration on random subsets of 12, 9 and 6 of the 15 participants who pressed, 20 subsets at each size. Specificity held at every size: at least 19 of 20 reference designs were certified, and 118 of 120 designs carried a genuine state. Sensitivity to an act-shaped component of 0.1 held at 14 of 20 with 12 participants (about 53 events) and 12 of 20 with 9 (about 40 events), and fell to 3 of 20 with 6 (about 26 events). The 95th percentile of the statistic over sound designs, which is the threshold a corpus of that size would calibrate, stayed between 0.066 and 0.095, below the adopted 0.10 at every size, so the threshold is conservative across the range tested.

The extended calibration (10 replicates per condition; Table S18) holds the certificate to departures from both shapes. Where preparation began on the round before the report, the second displacement step was material and the certificate moved the lead to two rounds or beyond in 18 of 20 designs, so that the estimate was read beyond the preparatory component (mean pooled ROC-AUC 0.493 at L = 2, against 0.730 and 0.734 at L = 1); with heavy-tailed act magnitudes the certified lead was one round. Across these three conditions, MTEP concluded that a detector works in 0 of 30 corpora containing no state. Persistent interference is a different case, because it is not confined to any unit the lead can step over: a shift that begins at the report reaches only the controls drawn after a report, since case windows end before it. At 0.1 local standard deviations, it left the estimate at chance (0.496; 0 of 10 false conclusions). At 1.0, the size of the genuine state of Table 5, it raised discrimination from later controls (mean 0.551 for a sustained shift and 0.552 for one decaying over five rounds) but not from earlier ones (0.495 and 0.500), and MTEP concluded that a detector works in 1 and 2 of 10. The discrimination of cases from earlier controls alone, which no post-report effect can reach, is therefore the check for persistent interference; it stayed between 0.599 and 0.804 for the genuine states, and on CogBeacon it was 0.619 (95% CI 0.551–0.712) for all features and 0.651 (0.579–0.752) for task telemetry (Section 3.7), so the CogBeacon estimate holds where post-report interference cannot reach.

Genuine states with non-Gaussian profiles were passed: across the abrupt sustained onset, the linear ramp, the asymmetric profile and heavy-tailed magnitudes, the certificate granted L* = 0 or 1 in 38 of 40 designs and MTEP concluded that a detector works in 37 of 40. In 2 designs, no lead within the sweep was certified, and in 1 the interval at the reported lead included 0.5, so MTEP withheld the verdict, which is the conservative error. Under label noise the certificate passed in 30 of 30 designs, and the verdict was retained when the report was displaced from the state by up to three rounds or when as many state episodes went unreported (20 of 20); the mean pooled ROC-AUC at one round was 0.661 and 0.671 under these two, and 0.536 when half of the reports carried no state, against 0.714 for the same state with every report carrying it (Table 5), and in that last condition the verdict was reached in 2 of 10. Spurious reports therefore cost power, as any target diluted by reports without a state must, while the certificate continued to pass; no form of label noise led MTEP to a false conclusion.

Table 6 reports the position certificate. With the design’s own sampler it certified 40 of 40 replicates, a specificity of 100% (95% CI 91–100%), at a mean position-only ROC-AUC of 0.493, the value the real design returns. Imbalance was detected in 14 of 40 replicates when a quarter of controls were drawn from the nearest earlier window, in 38 of 40 at one half and in 40 of 40 beyond, as the mean signed offset of controls moved from +0.35 to −5.58 rounds and the position-only ROC-AUC from 0.493 to 0.564, close to the 0.554 an unbalanced sampler produced while this design was being developed.

The linear position model cannot see an imbalance that leaves the mean offset unchanged, and the dispersion sweep measures what the nonlinear models add (Table S17). With the design’s own sampler the revised certificate was granted in 10 of 10 replicates. As controls were pushed away from their case, the mean absolute offset rose from 9.2 to 43.9 rounds while the mean signed offset stayed between −2.90 and +0.34; the linear model detected the imbalance in 0 of 10 replicates at γ_far = 1, whereas the spline, forest, boosting and nearest-neighbour models each detected it in 10 or more, and the revised certificate in 10; at γ_far = 0.5 the linear model detected it in 0 of 10 and the revised certificate in 10. The nonlinear baselines therefore extend the certificate to position dependence that the linear test cannot represent, and they are part of the certificate as revised.

**Figure 2:**
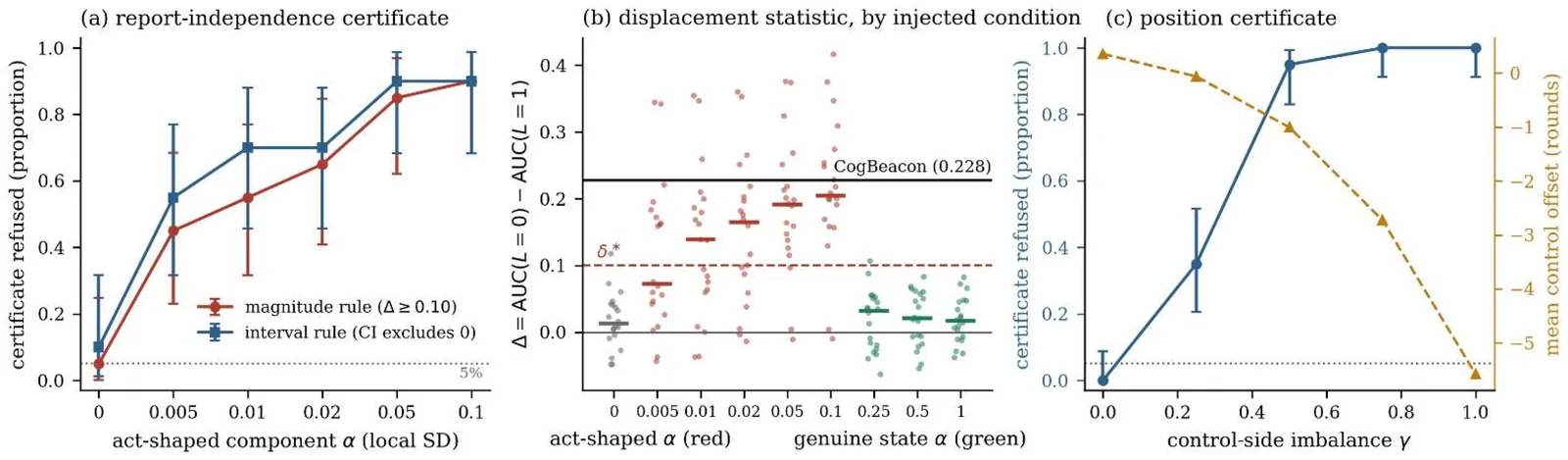
Calibration of the two certificates: (**a**) the report-independence certificate against artefact amplitude, (**b**) the displacement statistic by injected condition, (**c**) the position certificate against control-side imbalance. * The materiality threshold δ* = 0.10.

γ is the probability that a control is taken from the nearest remaining window before its case instead of by the design’s balancing rule, so γ = 0 is the design as specified and γ = 1 always takes the nearest earlier window. The certificate is granted when the participant-clustered bootstrap 95% interval for the position-only pooled ROC-AUC includes 0.5; the last column is the Clopper–Pearson 95% interval for the proportion certified. Figure 2 shows all three results together.

In (a) and (c), the vertical axis is the proportion of replicates in which the certificate is refused, with Clopper–Pearson 95% intervals, and (a) shows both decision rules. In (b) each point is one replicate: grey for the reference design, red for an act-shaped component of the stated amplitude, green for a genuine state of the stated amplitude. Bars mark medians, the dashed line is the materiality threshold and the solid line the value observed on CogBeacon. The right-hand axis of (c) carries the mean signed offset of controls.

### 3.3. Do the Four Designs Reach the Right Conclusion?

An evaluation design cannot be shown to be better by scoring higher, because a design that removes inflation scores lower. What can be shown is whether it reaches the correct conclusion on data whose truth is known, which the construction of Section 2.5 supplies. We therefore apply four designs to the same simulated corpora, each adding one property to the one above it, with the same estimator, features and verdict rule: whether the 95% interval for the pooled ROC-AUC excludes 0.5.

Table 4 (panel A) reports 20 replicates in each of four worlds. With nothing injected, the four designs withheld the verdict in 20, 17, 16 and 19 replicates; the shortfall from nineteen in twenty in the two grouped designs that read the estimate on the report is the slight anticonservatism of a bootstrap interval on twenty participants (Section 3.2). The decisive world is the second, where an act-shaped component is present and no state is. Random folds concluded that a detector works in 16 of 20 replicates, grouped leave-one-participant-out in 19 of 20, and grouped cross-validation with position-balanced matching, which holds three of the four properties and is the strongest design in current use, in 18 of 20, at mean pooled ROC-AUCs of 0.65, 0.70 and 0.70 for corpora containing no state whatever. MTEP concluded that a detector works in 1 of 20, at a mean of 0.52.

Specificity bought by discarding real signal would be worthless, so the third and fourth worlds test the converse. Where a genuine state was present, MTEP concluded correctly in 40 of 40 replicates, as did grouped cross-validation with matching in 40: nothing was lost by reading the estimate at a lead of one round. The certified lead L* was zero in 40 of 40, the certificate finding no material discontinuity, and the estimate was read at one round as Definition 1 requires; in the fourth world MTEP’s estimate was 0.70, as with the state alone (0.70), so the act was correctly judged immaterial. Across the four worlds, MTEP returned the correct verdict in 78 of 80 cases, against 62, 58 and 58 for the three designs above it.

The distance between the third design and the fourth is the contribution: the three properties that existing practice supplies are necessary and are assumed throughout, and MTEP shows that they do not imply the fourth. Section 3.5 shows the same gap on CogBeacon without simulation.

### 3.4. Position Balance Under the Conventional Protocol

**Figure 3:**
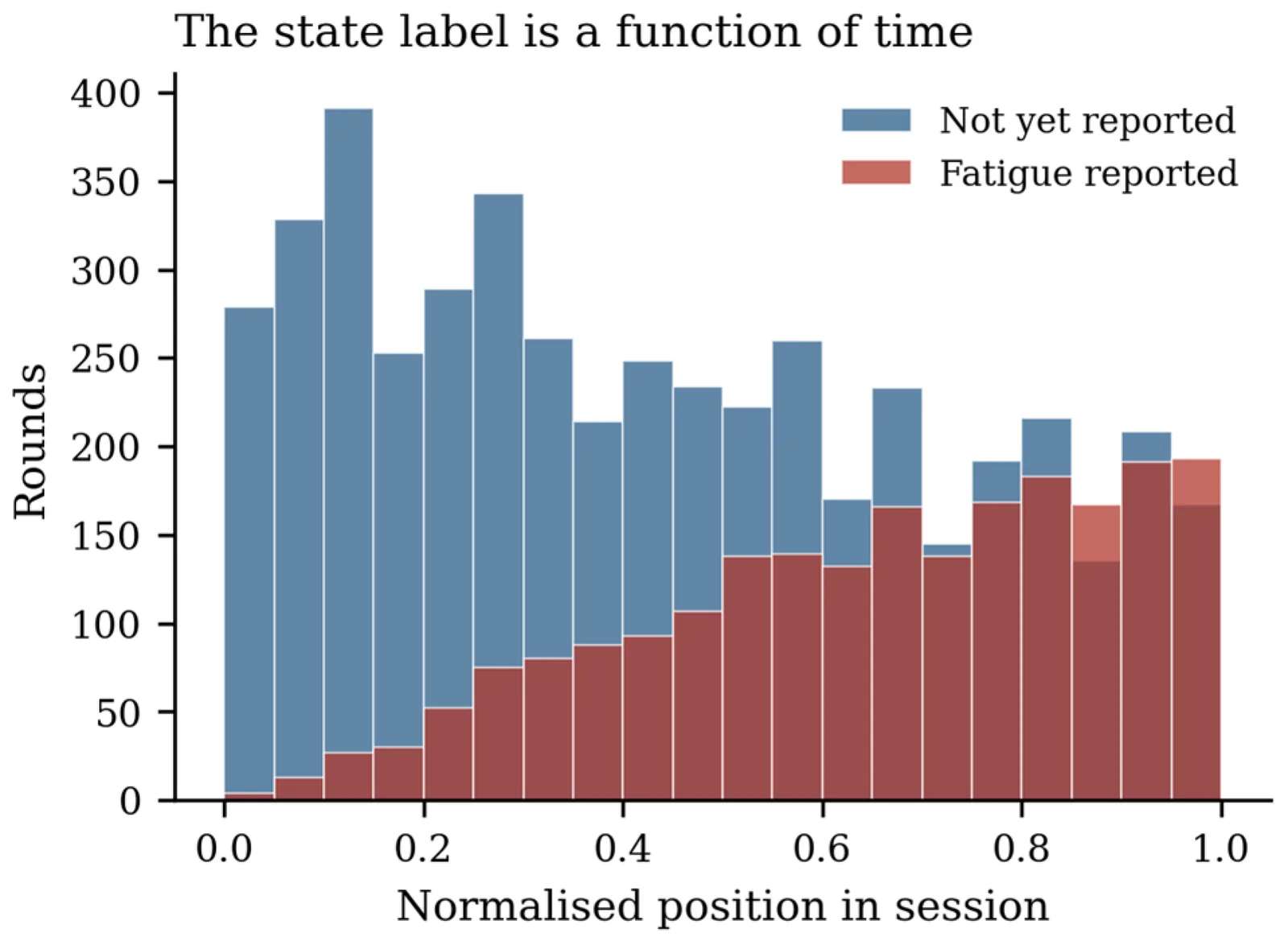
Session position and the cumulative-state label.

Table S8 reports all 14 Study 1 configurations. Under the cumulative-state label, the multimodal models reached pooled ROC-AUC between 0.678 and 0.767 and fold-wise ROC-AUC between 0.850 and 0.893, and among the modality ablations, task telemetry alone (0.721 pooled) outperformed EEG with facial features (0.538). A logistic regression given only the normalised round position reached a pooled ROC-AUC of 0.789 (95% CI 0.734–0.852; fold-wise 0.933 ± 0.054), and the deployable elapsed-rounds clock 0.774 (fold-wise 0.915 ± 0.067). On both summaries, the position-only model exceeds every multimodal configuration, including the Transformer, so a model with no access to EEG, facial behaviour or task performance outperformed every model that had such access (Figure 3).

Rounds are binned by normalised position within the session. Under the cumulative-state label, a round is positive once the participant has reported fatigue, so positives concentrate late in every session. The explanation is structural. Because the label is defined by a cumulative count, every round after the first press is positive and every round before it negative, so within a session position determines the label exactly. Matching does not repair this (Table S5): balancing positives and negatives within session-position deciles left the position-only model at a pooled ROC-AUC of 0.703 (95% CI 0.628–0.798) across the corpus and 0.894 (0.848–0.938) within participants, because position still orders the label within every decile and the 42 sessions with no press are uniformly negative, so session identity remains predictive. Position balance therefore cannot be established for the cumulative-state label within a session, which is why MTEP evaluates the event target.

### 3.5. Certifying Report-Independence on CogBeacon

**Figure 4:**
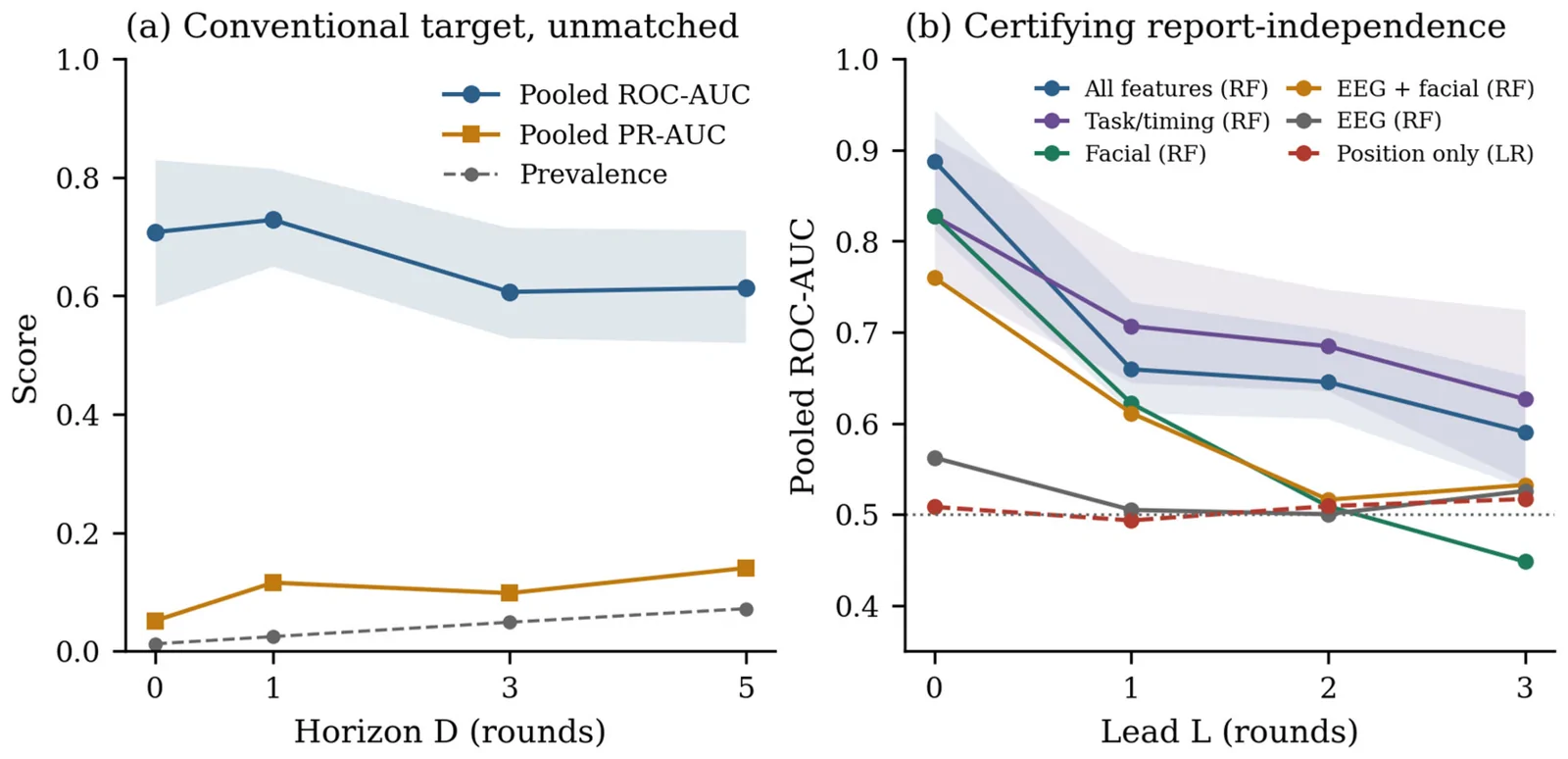
Two temporal sweeps: (**a**) the conventional event target against the horizon D; (**b**) the matched design against the lead L.

An event target admits position balance, and it is the setting in which report-independence has to be established. In Study 1 the event target at horizon D = 0, whose window ends on the press round, reached a fold-wise ROC-AUC of 0.872 ± 0.115, declining to 0.804, 0.748 and 0.704 at D = 1, 3 and 5; the pooled values (0.708, 0.728, 0.607, 0.614) are lower and less orderly, with overlapping confidence intervals (Figure 4a, Table S8). A decline with lead time is what a short-lived precursor would produce, and it is also what a trace of the press itself would produce. The matched design separates the two, and the certificate of Section 2.1.3 says which of them the estimate rests on.

(a) Study 1, all features, Transformer; at D = 0, the window ends on the press round, and the target moves further from the report as D grows. (b) MTEP step 6b; at L = 0, the case window contains the press round, and the certified lead of Table 7 is read from where the curve stops falling. Shaded bands: participant-clustered 95% CIs for all features and for task and timing features.

Table 7 and Figure 4b report the matched design as a function of the lead L. At L = 0, where every case window ends on the press round, all features reached a pooled ROC-AUC of 0.888, facial features alone 0.828 and EEG with facial features 0.760, while the position check stayed at chance (0.508); one round earlier these fell to 0.659, 0.622 and 0.611. A design that passes the position check can therefore still be dominated by the report. Facial features and EEG with facial features fell to chance by a lead of two rounds (0.509 and 0.516), whereas task telemetry declined gradually (0.707, 0.685 and 0.627 at L = 1, 2 and 3), the profile of an autocorrelated state rather than of a discontinuity.

On all features the displacement statistic is +0.228 from a lead of zero to one round (participant-clustered 95% interval +0.165 to +0.276), more than twice the materiality threshold and larger than in any of the 80 report-independent calibration cells, including those in which an injected genuine state produced discrimination of 0.714. One round further it is +0.014, well inside the threshold, so the design is certified report-independent at a lead of one round, where the analysis is read hereafter. The certified lead is the same for any materiality threshold between 0.06 and 0.22, a range containing every value from 0.075 to 0.20, so the conclusion does not rest on the choice of 0.10.

AUC values are pooled out-of-fold ROC-AUC of the matched design at leads of 0 to 3 rounds, from identical folds and estimators, under the rule calibrated in Table 5. Each displacement step carries a participant-clustered bootstrap interval, reported in the text. The certified lead L* is the smallest lead beyond which every further unit of displacement changes nothing material, at a threshold of 0.10; estimates are reported at max(1, L*) (Section 2.1.3). The same leads are reported on a fixed cohort of cases and controls (Table S12).

The certified lead differs by modality as the temporal argument predicts. EEG alone is certified at zero (first step +0.057), as expected of a lightweight headset that a button press barely disturbs, and is reported at L = 1, the smallest lead Definition 1 allows; task and timing telemetry at one round (+0.121), since the press lengthens the round it falls in; and facial features at two (+0.206, then +0.113). The press round is distinctive in the raw features: its median maximum landmark–centroid displacement is about twenty times that of other rounds and its median frame count, and hence duration, was 44% higher (Table S7), consistent with the participant reaching for the button, looking away and taking longer to answer. The step size thus measures how much of a stream is mechanically coupled to the act, which is why the primary analysis is reported on all features at the lead at which they are jointly certified.

Preparation for the press begins before the press round, and the certificate detects it. In the round before a press, the median maximum landmark–centroid displacement was 3.9 times, and the median mean displacement 3.3 times, that of rounds more than five rounds from any press, while round duration was one frame (11%) above its usual value and response time unchanged; two rounds before a press none of the four was elevated by more than 11% (Table S11). This is the trace that moves the certified lead of facial features to two rounds: Definition 1 excludes the press round, and the second displacement step excludes the round of preparation before it.

Because the sweep rebuilds the matched design at each lead, the cases and controls entering adjacent leads can differ. We therefore repeated it on a fixed cohort: the 63 presses whose case windows are admissible at every lead from 0 to 3, each with the same three controls at every lead (189 controls, 15 participants), so that only the case window moves (Table S12). Every reported lead was unchanged. On all features the first step was +0.205 (95% CI +0.112 to +0.284) and the second +0.033 (−0.033 to +0.094), certifying at one round, where the pooled ROC-AUC was 0.690 (0.635–0.760); facial features stepped by +0.212 and +0.162 before a step of +0.003, certifying at two rounds; EEG showed no material step. Task and timing features stepped by +0.094 and +0.066, so they are reported at one round, as in Table 7, with a pooled ROC-AUC of 0.730 (0.654–0.821). The protocol therefore reports every feature set at the same lead whether or not the cohort is held fixed.

### 3.6. Controlled Analysis Under MTEP

Table 8 reports Study 2 at L = 1. The position check passes: the position-only model reached a pooled ROC-AUC of 0.493 (95% CI 0.482–0.512; fold-wise 0.524 ± 0.098), so session position carries no usable information about case status in the matched sample. Nonlinear position models agree. A cubic spline, a random forest, gradient boosting and 25-nearest neighbours on the same position summary returned 0.489, 0.533, 0.507 and 0.533, and a random forest and gradient boosting on a position family that adds question index, elapsed rounds and session length returned 0.554 and 0.557, every 95% interval including 0.5 (Table S16).

With both properties established, a signal significantly above chance remains. The random forest on all features reached a pooled ROC-AUC of 0.659 (95% CI 0.612–0.733; fold-wise 0.759 ± 0.196), and on task and timing features alone 0.707 (95% CI 0.645–0.789). EEG with facial features reached 0.611 (95% CI 0.551–0.672), facial features alone 0.622 (95% CI 0.537–0.694) and EEG alone 0.505 (95% CI 0.456–0.557). Pooled precision–recall AUC for all features was 0.412 against a prevalence of 0.25 (Figure S2). The estimate does not rest on a few participants: removing any single participant left the all-feature pooled ROC-AUC between 0.647 and 0.674 (task telemetry 0.691 to 0.736), and the model ranked cases above controls within 14 of the 15 participants (Table S1). Operating points are evaluated on the full recording stream (Section 3.10).

Control-side variants show what bilateral matching secures (Table S15). Drawing every control before its case, or every control after it, makes position a predictor again: the position-only model rose to 0.583 (95% CI 0.577–0.603) and 0.653 (0.628–0.680), so both variants fail the position certificate and compare cases with windows at a systematically different place in the session. The bilateral design is the variant in which P3 holds. Task telemetry discriminated cases under all three, at 0.696, 0.704 and 0.818 with earlier, bilateral and later controls, and the excess with later controls follows the position imbalance of that variant (paired by participant against the bilateral design, wins/ties/losses 9/5/1, *p* = 0.014; earlier controls 6/3/6, *p* = 0.970). Requiring that no two windows of a set share a round gave 0.708 for all features and 0.735 for task telemetry, against 0.661 and 0.704 in the bilateral design (paired by participant, *p* = 0.620 and 0.638), so the estimate holds with no overlap between windows. That variant fails the position certificate (0.545, 0.516–0.589), because excluding overlapping candidates leaves controls unevenly placed around their case, which is why the design retains overlap within a set.

### 3.7. Modality Contributions

The ablations locate the remaining signal in task telemetry. Task and timing features alone matched the full feature set (fold-wise 0.776 against 0.759; paired test Holm *p* = 1.000, wins/ties/losses 4/5/6), and both exceeded the position baseline significantly (task: Holm *p* = 0.018; all features: Holm *p* = 0.026). EEG alone was at chance. EEG with facial features did not significantly exceed the position baseline (Holm *p* = 1.000) or EEG alone (Holm *p* = 0.383) and was not better than facial features alone (Holm *p* = 1.000, wins/ties/losses 6/1/8). All paired tests are in Table S3, and per-participant results in Table S1 and Figure S1.

Task telemetry therefore carries most of what can be detected one round before a report: response time, errors and running accuracy in the preceding rounds. Participants were instructed to press when having difficulty keeping up, and MTEP isolates the measurable precursor of that report: performance declines in the rounds before a participant reports difficulty, separated from the report itself by the certified lead.

Four analyses identify what that precursor is (Tables S13 and S14). Removing the two position-like variables, normalised round position and question index, left task telemetry at a pooled ROC-AUC of 0.701 (95% CI 0.642–0.787) and all features at 0.662 (0.622–0.727); removing difficulty level as well left 0.697 (0.633–0.782). The eight performance variables alone (score, response time, correctness, perseveration, the two error increments, response-time deviation and running accuracy) reached 0.695 (0.638–0.782) at one round and 0.696 (0.638–0.768) at two, whereas round duration alone was at chance (0.546, 0.456–0.622). Cases were discriminated both from the controls that preceded them (task telemetry 0.651, 0.579–0.752) and from those that followed them (0.813, 0.719–0.902). Time-on-task drift would raise discrimination from earlier controls but lower it from later ones, and a post-report effect can reach only later controls, so discrimination above chance on both sides is produced by neither alternative alone. The precursor is a decline in performance, not the scheduled difficulty of the task, the clock, the lengthening of the press round or drift across the session. (Revision analyses were run with scikit-learn 1.9.1, which reproduces the Table 8 estimates to within 0.005.)

### 3.8. Permutation Null and Model Comparisons

Under the matched-set permutation null, in which case status was permuted within each matched set and the full cross-validation repeated 200 times for the same random-forest models, the observed pooled ROC-AUC for all features of 0.661 exceeded a null distribution with mean 0.497 and 95th percentile 0.556, and the observed 0.610 for EEG with facial features exceeded a null with mean 0.495 and 95th percentile 0.560. Both observed values exceed all 200 null values (*p* = 0.005 in each case), lying 0.105 and 0.050 above the null 95th percentiles; the null was run on a separate machine, and its observed values differ from Table 8 in the third decimal. The EEG-plus-facial result therefore exceeds chance at L = 1, but EEG alone was at chance from L = 1 onward, facial features alone performed similarly (0.622), and the EEG-plus-facial result fell to chance at L = 2 (Section 3.5); we read it as a facial signal confined to the round before the press. The permutation test establishes whether a pooled result exceeds chance under the matched structure; the magnitude of the effect is given by the intervals in Table 8.

The Transformer did not outperform the classical models. Under the conventional protocol its fold-wise ROC-AUC was 0.850 against 0.868 for logistic regression; under the controlled design its pooled ROC-AUC was 0.610 against 0.659 for the random forest (Holm *p* = 1.000, wins/ties/losses 6/4/5). Of the family of 10 paired comparisons (Table S3), two survive Holm correction, all features and task telemetry each over the position-only model; no comparison between feature sets or between model families does. Under identical folds, features and settings, the Transformer therefore offered no advantage over the random forest at this sample size. With 68 events, fold-wise standard deviations near 0.2 and several participants contributing one or two cases, a strong classical baseline, pooled confidence intervals and a paired test across participants should be preconditions for claiming an architectural advantage on data of this kind.

### 3.9. Sensitivity of MTEP to Its Free Parameters

Table S2 reports the 33 configurations at L = 1. The position check comes first. Its pooled ROC-AUC ranged from 0.444 to 0.536, but because a single-variable model’s out-of-fold scores are nearly deterministic, its bootstrap interval is narrow, and in 16 configurations the interval excluded 0.5: all nine with a five-round window, all three with a ten-round gap at W = 10 and at W = 15, and the one-control design. Under the protocol, these fail the position check and are not interpreted, which also shows that the check is not a formality: small changes to the design can reintroduce a detectable position imbalance. In the 17 that pass, the all-feature random forest had a median pooled ROC-AUC of 0.659 (range 0.616 to 0.703; interval above 0.5 in 17 of 17), task telemetry 0.707 (0.656 to 0.739; 17 of 17) and EEG with facial features 0.580 (0.494 to 0.619; 11 of 17), and the same ordering holds across all 33. Within this grid the qualitative picture does not depend on the choice of window, gap, offset, control ratio or seed. Of the three construction parameters, the maximum offset had no effect at all: every estimate was identical at 20, 30 and 50 rounds, because controls are drawn from the nearest eligible windows. Window length and minimum gap carry the design: they decide whether the position certificate passes, every five-round window and every ten-round gap failing it, and they move the all-feature estimate by up to 0.05 in median, so they are the two settings to choose for a new corpus, with the position certificate identifying an unsuitable choice.

The grid holds the lead fixed; the certificate itself, a difference in pooled ROC-AUCs with the lead counted in rounds, was tested separately against the estimator and the window length. Repeating the displacement sweep with each of the three classical estimators, and with the random forest at window lengths of 5, 10 and 15 rounds (Table S9), all six runs certify at a lead of one round. The first displacement step ranges from +0.194 to +0.258 across them, always more than twice the materiality threshold, and the second from −0.024 to +0.043, always well inside it. The estimators disagree about the level, as they should; logistic regression reaches 0.602 at the certified lead against 0.659 for the random forest and 0.664 for the SVM, but none changes where the estimate stabilises: the certificate reads a property of the design, not of the analyst’s choices within it.

### 3.10. Performance on the Full Recording Stream

Table 9 reports the certified detector on the full recording stream: 6202 windows from 20 participants, of which 77 (1.2%) preceded a press. The ranking learned on the matched sets carried over to the stream. Task and timing telemetry reached a pooled ROC-AUC of 0.686 (95% CI 0.604–0.781) and all features 0.629 (0.557–0.724), against 0.707 and 0.659 on the matched sets, while the position-only model stayed at chance (0.509). Alerting on the 5% of windows that task telemetry scored highest gave a precision of 4.8%, 3.9 times the base rate, and flagged 19% of presses one round ahead and 32% within three rounds; at a 10% budget these rose to 36% and 44%. The certified detector therefore concentrates upcoming reports into a small share of the recording, and the stream evaluation supplies the quantity a deployment decision needs: how often an alert precedes a report.

Models are trained as in Study 2 and reproduce its matched-set ROC-AUC (Table 8) exactly. Alert budgets rank all windows of all held-out participants by score; a press counts as flagged within three rounds when any of the three windows ending one to three rounds before it is alerted.

### 3.11. Application to a Second Corpus

To test the design on a different act, label and sensor set, we applied MTEP to CL-Drive [52]. Twenty-one drivers completed nine induced complexity levels of 3 min each in a driving simulator while EEG (four-channel Muse), EDA and gaze were recorded and spoke a 1–9 cognitive-load rating every 10 s in response to an audio cue, the spoken-rating row of Table 2 (ECG was not used). The act is visible in the sensors: power above 45 Hz at the temporal electrodes rose from 1.5 to 4.5 s after each cue, so the 10 s segments used in the published evaluation contain the previous spoken report. Each unit was therefore summarised over 3 s windows placed relative to the report, from a window covering the report itself to report-free windows ending 1.0 s after, and 0.5 and 2.0 s before, the cue, with features expressed relative to each level’s pre-drive baseline recording (Supplementary Section S18).

Under the published protocol (high load for ratings 5–9, leave-one-participant-out), a clock given only the level and the segment index reached an accuracy of 73.7% (ROC-AUC 0.741, 95% CI 0.693–0.812), against published accuracies of 70.70%, 73.01% and 74.68% for EEG, EEG with EDA and EEG with gaze [52]. As on CogBeacon (Section 3.1), the published subject-independent figures are within reach of a variable that carries no sensor information. Under the published ten-fold protocol, our untuned forest reached 75.6% to 82.5%; with its settings selected by nested cross-validation inside each training fold, it reached 80.4% for EEG with EDA and 81.0% for EEG with gaze, above the published 80.06% and 80.75%, and 76.4% for EEG against 77.41%. Table S19 reports every configuration tried, including subject-independent evaluation with participant-wise normalisation.

Every unit carries a spoken report, so the act is present for both label values, and the certificate should find nothing to remove. It found nothing: including the spoken report in the window left discrimination of the rating unchanged (pooled ROC-AUC 0.582 with the report and 0.593 report-free), and for the event target, a rating higher than the previous one (93 cases with 186 bilateral controls), the first displacement step was +0.090 (95% CI −0.022 to +0.191), below the materiality threshold, so L* = 0 in every modality. Proposition 1 needs an act that occurs with one label value and not the other; a rating spoken at every unit carries the label only through the content of the act, and the certificate measures that route as immaterial here.

Position balance decides the event target. Reported increases cluster early in each level, and bilateral matching did not remove that dependence: the linear position model returned 0.547 (95% CI 0.522–0.568), the spline, forest, boosting and nearest-neighbour models 0.608 to 0.650, and the position family with the level 0.731 (95% CI 0.651–0.807). MTEP refuses P3 for this target and withholds a verdict; the nonlinear models register an excess over chance of 0.108 to 0.150, 2.3 to 3.2 times that of the linear model.

The induced level supplies a criterion independent of the report. Decoding high (7–9) against low (1–3) levels from report-free windows under leave-one-participant-out reached 0.926 (95% CI 0.903–0.947) with all sensors and 0.809 with EEG alone, while the segment index was uninformative (0.500); from the same windows the self-reported rating reached 0.593. The sensors track the experimentally induced load strongly, and the self-reported rating follows the induced condition and each participant’s use of the scale, which is what the clock exploits.

## 4. Discussion

Table 10 sets each claim of the paper beside the result that supports it.

### 4.1. What the Certified Guarantees Add to a Reported Accuracy

Stating P3 and P4 as design properties and certifying them gives a reported accuracy a defined meaning at very little cost. Under the cumulative-state label, a single variable encoding position within a session was competitive with every multimodal model tested, so an accuracy obtained under that labelling cannot by itself separate fatigue from session duration. Section 3.1 settles this for the published CogBeacon results: under the dataset paper’s own protocol, the clock exceeded the published ensemble on accuracy and macro F1. One additional number, costing seconds to compute, settles the question for any future result. Because position balance is a property of the target definition rather than of the architecture, the windowing or the cross-validation scheme, no later modelling choice can supply it; corpora that timestamp individual reports, as CogBeacon does, support event targets that MTEP evaluates directly.

### 4.2. What the Report-Independence Certificate Adds

The report-independence certificate turns P4 from an assumption into a measurement. Because its calibration supplies a specificity and a sensitivity, the displacement step of +0.228 observed on CogBeacon is a quantified result: an injected component of 0.05 local standard deviations, whose mean displacement of +0.188 lies below the +0.228 observed, was caught in 30 of 40 calibration replicates, and a genuine state of comparable strength was passed. The certificate locates the lead at which the estimate stabilises, and the size of the step measures the instrumentation itself: it was largest for facial features and smallest for EEG, ordering the streams by how much of the reporting act they record. Each property therefore needs its own certificate, because holding one says nothing about the others (Section 3.5).

### 4.3. Applying MTEP to Other Corpora

Whether the requirement binds can be read from a data statement before any modelling: how the label was produced, whether windows can overlap the act, and whether any recorded channel responds to it. Table 2 gives the answer for the mechanisms in common use, and for a new collection, recording the time of the reporting act separately from the report makes both the property and its certificate available. The design applies wherever a label is assigned to, or accumulates within, a recording unit and samples are drawn more finely than labels. The lead and the materiality threshold are corpus-specific, and the released implementation recalibrates both on request; the calibration by corpus size shows that the certificate keeps its specificity at every size tested and its sensitivity down to about 40 events from nine participants.

### 4.4. What Signal Remains and Where It Comes from

Under both certificates, a significant signal remains (pooled ROC-AUC 0.659, permutation *p* = 0.005), carried mainly by task performance in the rounds before a report and retained on the full recording stream. The modality attribution changed as each property was secured, which shows what the design delivers. Without position balance, task telemetry appeared to carry the signal, because it contains the clock. With position balance but without report-independence, EEG and facial behaviour appeared to carry it, because they register the report. With both properties in place, a stable attribution emerges: participants who report difficulty keeping up have, shortly before, been performing worse. Securing the properties therefore does more than adjust the headline figure; it decides which modality a study is about.

The certified estimate answers a precise question. Performance declines measurably in the rounds before a participant reports difficulty keeping up, and the sensors detect that decline one round ahead, independently of the act of reporting and of the clock. CogBeacon operationalises cognitive fatigue as that report, so the estimate is a fatigue estimate to the extent that the report is a fatigue measure; a corpus recording an independently validated fatigue measure alongside in-task reports would allow the certified estimate to be read against it directly, and MTEP applies to it unchanged. Label noise in the report lowers the attainable estimate but leaves MTEP’s verdicts intact (Section 3.2).

### 4.5. Limitations and Future Work

The controlled analysis rests on 68 events from 15 participants; its primary interval (0.612–0.733) excludes chance, removing any one participant leaves the estimate between 0.647 and 0.674, and the calibration by corpus size shows that the certificate remains sensitive down to about 40 events. CL-Drive (Section 3.11) applies the design to a spoken report, a graded label and a different sensor set, and the calibration on corpora of known truth varies the shape, persistence and noise of both the act and the state (Section 3.2 and Section 3.3); a corpus pairing in-task event reports with an independently validated state measure would extend both. A reporting act whose behavioural consequence persists for the rest of a session lies outside what a displacement step can key on; MTEP covers it with the discrimination of cases from earlier controls alone, which such a consequence cannot reach (Section 3.2). Applying MTEP to further benchmarks that meet the conditions of Table 2, extending the certificates to other routes by which label information could reach the features, and tuning models under the certified design are the next steps.

## 5. Conclusions

On the same EEG features, protocols and metrics as the published CogBeacon studies, our model exceeds every published single-classifier accuracy, and the same comparison shows that those accuracies are set by the evaluation design: a sensor-free clock exceeds the dataset paper’s result, and under random splits, windowed rows share rounds across the split. MTEP supplies the guarantees under which an accuracy can be read as detection of the reported state rather than of the clock or of the report. MTEP is an evaluation design for learning from labels that participants produce during the recording. It adopts the three properties that existing designs supply and adds report-independence, a timing property none of them implies, with calibrated certificates for both position balance and report-independence. On simulated corpora of known truth it avoided the false conclusion that designs in current use reached in 16 to 19 of 20 cases, without losing a true positive, and on CogBeacon it yields an interpretable estimate at a certified lead of one round, pooled ROC-AUC 0.659, carried mainly by task performance before a report. Applied to the full recording stream, the certified detector retains its ranking (ROC-AUC 0.686 for task telemetry at a prevalence of 1.2%). On CL-Drive, a corpus with spoken ratings, a sensor-free clock matched the published subject-independent accuracies, the report-independence certificate stayed silent where the act was common to both labels, and the position certificate refused a target whose reports cluster within each drive.

Report-independence has, to our knowledge, not previously been stated as a requirement on an evaluation design, nor decided by a calibrated test. Three things follow for anyone working with labels of this kind: Before modelling, check the corpus against the four properties, which Table 2 does for the label-production mechanisms in common use. Where report-independence has to be engineered, report the displacement statistic beside the headline figure, and report a position-only baseline whenever the target accumulates within a recording. And when designing a collection, record the timestamp of the reporting act separately from the report, since that single field is what makes the property available at analysis time. We release the design, both certificates, their calibration, the derived feature matrices and the per-fold predictions, so that the guarantees can be reproduced and recalibrated on other corpora.

## Figures and Tables

**Table 1:** The four properties: what each controls, what it leaves open, how MTEP secures it and how it is verified.

Property	What It Controls	What It Leaves Open	How MTEP Secures It	How It Is Verified
P1 label and feature separation	Which streams may produce a feature	What the sensor streams themselves contain	Disjoint label and feature pipelines sharing no state	A closing assertion at build time on every feature name
P2 participant-level generalisation	Whose data appear in training and in evaluation	Structure within a single participant’s own recording	Grouped leave-one-participant-out cross-validation	Fold membership: no participant appears on both sides
P3 within-recording position balance	Where a window lies inside its recording	What that window contains	Matched sampling with control windows balanced before and after each case	Position certificate: the 95% interval for the pooled AUC of every position-only baseline, linear and nonlinear, must include 0.5
P4 report-independence	What a window contains at the moment the label is made	How much genuine signal surrounds the report	Case windows end L units before the reported unit; no window spans one	By construction, every estimate is read at a lead of at least one unit; the report-independence certificate, the displacement statistic D(L), locates the smallest lead L* beyond which every further step is immaterial, and the estimate is reported at max(1, L*)

**Table 2:** When report-independence must be engineered.

Label-Production Mechanism	Example	C1	C2	C3	Must Be Engineered
In-task button or key press	CogBeacon [1]; in-vehicle drowsiness buttons	Yes	Yes	Yes	Yes
On-screen slider or rating completed during the session	Mid-task workload probes	Yes	Yes	Yes	Yes
Spoken rating during recording	Think-aloud and verbal workload protocols	Yes	Yes	Yes	Yes
Questionnaire at a block or session boundary	Chalder scale [44] in MEFAR [45]; FatigueSet [2]	Yes	Only if features run up to the questionnaire	Yes	Depends on windowing
Retrospective questionnaire after the recording ends	End-of-shift surveys	Yes	No	Yes	No
External observer rating or video coding	Expert-annotated drowsiness	No	--	--	No
Instrumented behavioural criterion	Lapses in a psychomotor vigilance task [5]	No	--	--	No (see text)
Independent physiological criterion	Polysomnography-scored sleep stage	No	--	--	No

C1 to C3 are as defined in Section 2.1.2, with C2 judged under the windowing convention in common use. Report-independence must be engineered only when all three hold.

**Table 3:** Comparison with published results on CogBeacon: EEG features, with the protocol and metrics of the original study (cumulative fatigue label).

Study	Protocol	Metric	Published (EEG)	Ours (EEG)	Clock (No Sensor)
Papakostas et al. [1]	20% of sessions held out, 10 folds; balanced training	Accuracy/ macro F1	0.64/0.60 (single); 0.67/0.63 (ensemble)	0.668/0.590	0.686/0.637
Ekim et al. [50]	Random 50/50 split of rounds	Accuracy	83.14% (SVM); 83.34% (k-NN)	96.5%	68.8%
Das et al. [51]	Stratified 10-fold cross-validation, natural class ratio	Accuracy/ F1 (fatigue)	90.96%/0.86	98.5%/0.976	69.8%/0.582

**Table 4:** MTEP against baseline evaluation designs and baseline models. (A) Four evaluation designs on simulated corpora of known truth. (B) Baseline models under MTEP on CogBeacon at lead L = 1. L*: certified lead.

*(A) Evaluation designs on simulated corpora (20 replicates per world)*						
**Design**	**Properties**	**No Injection**	**Act Only**	**State Only**	**Act and State**	**Correct**
Random folds, unbalanced, on the report	P1	0/20 (0.44)	16/20 (0.65)	19/20 (0.67)	19/20 (0.69)	62/80
Grouped LOSO, unbalanced, on the report	P1, P2	3/20 (0.51)	19/20 (0.70)	20/20 (0.71)	20/20 (0.73)	58/80
Grouped LOSO, balanced, on the report	P1, P2, P3	4/20 (0.52)	18/20 (0.70)	20/20 (0.72)	20/20 (0.73)	58/80
MTEP, read at max(1, L*)	P1–P4	1/20 (0.52)	1/20 (0.52)	20/20 (0.70)	20/20 (0.70)	78/80
*(B) Baseline models under MTEP on CogBeacon (68 cases, 204 controls, 15 participants)*						
**Model**	**Features**	**Matched ROC-AUC [95% CI]**	**Matched PR-AUC**	**Reported Lead**	**Full-Stream ROC-AUC**	***p* (Perm.)**
Clock (LR)	Round position	0.493 [0.482, 0.512]	0.252	—	0.509	—
RF	EEG	0.505 [0.456, 0.557]	0.273	L = 1 (L* = 0)	—	—
RF	Facial	0.622 [0.537, 0.694]	0.411	L = 2	—	—
RF	EEG + facial	0.611 [0.551, 0.672]	0.320	L = 1	—	0.005
RF	Task and timing	0.707 [0.645, 0.789]	0.467	L = 1	0.686	—
LR	All	0.602 [0.541, 0.669]	0.387	L = 1	—	—
Transformer	All	0.610 [0.541, 0.692]	0.348	—	—	—
RF (ours)	All	0.659 [0.612, 0.733]	0.412	L = 1	0.629	0.005

**Table 5:** Calibration of the report-independence certificate, 20 replicates per condition.

Injected	Amplitude	AUC (L = 0)	AUC (L = 1)	D = AUC(L = 0) − AUC(L = 1)	Certified, Interval Rule	Certified, Magnitude Rule
Act-shaped component	0 (reference)	0.538 (0.040)	0.521 (0.034)	+0.017 (0.042)	18/20	19/20
Act-shaped component	0.005	0.634 (0.097)	0.521 (0.034)	+0.113 (0.112)	9/20	11/20
Act-shaped component	0.01	0.654 (0.098)	0.521 (0.034)	+0.133 (0.112)	6/20	9/20
Act-shaped component	0.02	0.672 (0.094)	0.521 (0.034)	+0.151 (0.109)	6/20	7/20
Act-shaped component	0.05	0.709 (0.085)	0.521 (0.034)	+0.188 (0.102)	2/20	3/20
Act-shaped component	0.1	0.735 (0.087)	0.521 (0.034)	+0.214 (0.105)	2/20	2/20
Genuine cumulative state	0.25	0.630 (0.073)	0.611 (0.073)	+0.019 (0.046)	17/20	19/20
Genuine cumulative state	0.5	0.670 (0.072)	0.649 (0.058)	+0.020 (0.042)	16/20	20/20
Genuine cumulative state	1	0.733 (0.054)	0.714 (0.051)	+0.019 (0.035)	15/20	20/20

**Table 6:** Calibration of the position certificate, 40 replicates per condition.

Gamma	Mean Signed Offset (Rounds)	Fraction of Controls After Case	Position-Only AUC	Designs Certified	95% CI
0.00	+0.35	0.34	0.493 (0.003)	40/40	0.91–1.00
0.25	−0.07	0.32	0.502 (0.007)	26/40	0.48–0.79
0.50	−1.01	0.27	0.516 (0.006)	2/40	0.01–0.17
0.75	−2.73	0.18	0.536 (0.005)	0/40	0.00–0.09
1.00	−5.58	0.04	0.564 (0.000)	0/40	0.00–0.09

**Table 7:** Certifying report-independence on CogBeacon. L*: certified lead.

Feature Set	L = 0	L = 1	L = 2	L = 3	D (0 to 1)	D (1 to 2)	D (2 to 3)	Certified Lead
All features	0.888	0.659	0.645	0.590	+0.228	+0.014	+0.055	L = 1
EEG + facial	0.760	0.611	0.516	0.532	+0.148	+0.095	−0.016	L = 1
EEG only	0.562	0.505	0.500	0.526	+0.057	+0.005	−0.026	L* = 0; reported at L = 1
Facial only	0.828	0.622	0.509	0.448	+0.206	+0.113	+0.061	L = 2
Task/timing only	0.827	0.707	0.685	0.627	+0.121	+0.022	+0.058	L = 1

**Table 8:** Study 2, MTEP within-session case–control design at L = 1 (68 cases, 204 controls, 34 sessions, 15 participants). Pooled values with participant-clustered bootstrap 95% CI; fold-wise values mean ± SD.

Configuration	Pooled ROC-AUC [95% CI]	Pooled PR-AUC	Fold ROC-AUC	Fold Bal. Acc.
Position only (LR) (position certificate)	0.493 [0.482, 0.512]	0.252	0.524 ± 0.098	0.512 ± 0.040
All features (RF)	0.659 [0.612, 0.733]	0.412	0.759 ± 0.196	0.520 ± 0.084
All features (LR)	0.602 [0.541, 0.669]	0.387	0.711 ± 0.179	0.609 ± 0.152
Task/timing only (RF)	0.707 [0.645, 0.789]	0.467	0.776 ± 0.189	0.675 ± 0.194
EEG + facial (RF)	0.611 [0.551, 0.672]	0.320	0.569 ± 0.219	0.489 ± 0.026
EEG + facial (LR)	0.535 [0.482, 0.607]	0.327	0.591 ± 0.207	0.576 ± 0.143
Facial only (RF)	0.622 [0.537, 0.694]	0.411	0.644 ± 0.223	0.554 ± 0.192
EEG only (RF)	0.505 [0.456, 0.557]	0.273	0.492 ± 0.123	0.500 ± 0.000
All features (Transformer)	0.610 [0.541, 0.692]	0.348	0.684 ± 0.243	0.585 ± 0.179

**Table 9:** The certified detector on the full recording stream at L = 1 (6202 windows, 20 participants, 77 windows preceding a press; prevalence 0.012).

Model	ROC-AUC [95% CI]	PR-AUC	Precision at 5% (Lift)	Presses Flagged at 5% (Next/Within 3)	Precision at 10% (Lift)	Presses Flagged at 10% (Next/Within 3)
All features (RF)	0.629 [0.557, 0.724]	0.026	3.9% (3.1×)	16%/23%	3.5% (2.9×)	29%/34%
Task/timing only (RF)	0.686 [0.604, 0.781]	0.030	4.8% (3.9×)	19%/32%	4.5% (3.6×)	36%/44%
Position only (LR)	0.509 [0.471, 0.551]	0.014	1.6% (1.3×)	6%/6%	1.3% (1.0×)	10%/10%

**Table 10:** Claims and the evidence for them.

Claim	Evidence	Where
Report-independence is a requirement that existing designs do not imply	Designs holding P1–P3 concluded that a detector works in 16 to 19 of 20 corpora containing only the act; MTEP in 1 of 20. Correct verdicts 78/80 against 58–62/80	Table 4A; Section 3.3
The certificate is calibrated and specific	Specificity 39/40; act of 0.05 local SD refused in 30/40; all 80 designs with a genuine state of amplitude 0.5 or 1.0 certified	Table 5 and Table S10
Preparation for the report is located and stepped over	Simulated preparation: lead moved beyond it in 18/20 designs, 20/20 correct verdicts; CogBeacon: facial motion 3.9× usual in the round before a press, facial lead 2	Section 3.5; Tables S11 and S18
Verdicts hold under other temporal profiles and label noise	No false conclusion under heavy-tailed acts or any of three label-noise forms; 37/40 non-Gaussian states detected, the rest withheld	Section 3.2; Table S18
The position certificate sees nonlinear dependence	Dispersion imbalance: linear test 0/10, revised certificate 10/10, specificity 10/10; CL-Drive: nonlinear models register 2.3–3.2 times the linear excess	Tables S16, S17 and S20
The certified estimate is stable	Same leads on a fixed cohort of cases and controls; 17 of 17 position-balanced design configurations above chance; one lead across six estimator and window settings; 0.647–0.674 leaving out any participant	Tables S2, S9 and S12; Section 3.6
The signal is a decline in performance	Performance variables alone 0.695; round duration 0.546; above chance against both earlier (0.651) and later (0.813) controls	Section 3.7; Tables S13 and S14
Our model exceeds published accuracies on the same features and protocols	CogBeacon: 0.668 vs. 0.64, 96.5% vs. 83.3%, 98.5% vs. 91.0%; CL-Drive ten-fold: 80.4% vs. 80.06%, 81.0% vs. 80.75%	Table 3 and Table S19
Published accuracies follow the evaluation design, on two corpora	Sensor-free clock: 0.686 against the published 0.67 ensemble on CogBeacon; 73.7% against published 70.7–74.7% subject-independent accuracies on CL-Drive	Table 3; Section 3.11

## Data Availability

The CogBeacon dataset analysed in this study is publicly available at https://github.com/MikeMpapa/CogBeacon-MultiModal_Dataset_for_Cognitive_Fatigue (accessed on 10 September 2026), and the CL-Drive dataset at https://doi.org/10.5683/SP3/JJ2YZZ (accessed on 10 September 2026). The complete reproducibility recipe and accompanying code are publicly archived at https://github.com/fjeribi/mtep (accessed on 10 September 2026).

## Supplementary Materials

The following supporting information can be downloaded at https://www.mdpi.com/article/10.3390/diagnostics16213556/s1: Table S1: Per-participant ROC-AUC in the controlled design; Figure S1: ROC-AUC for each held-out participant; Figure S2: The matched design at L = 1 (control positions, ROC and precision–recall curves); Table S2: Complete sensitivity grid; Table S3: Complete paired tests; Table S4: Feature inventory; Table S5: Position-decile matching on the cumulative-state label; Table S6: Model settings; Table S7: Medians of selected per-round features in press and other rounds; Table S8: Study 1, all configurations; Table S9: The certified lead under other estimators and window lengths; Section S10: Calibration implementation details; Section S11 and Table S10: Calibration of the report-independence certificate by corpus size; Section S12: Contents of the code and data release; Section S13, Tables S11 and S12: Preparation before the press and the fixed-cohort lead sweep; Section S14, Tables S13 and S14: What the task signal is; Section S15 and Table S15: Control-side variants; Section S16, Tables S16 and S17: Nonlinear position baselines and the power of the position certificate; Section S17 and Table S18: The report-independence certificate under other temporal profiles, persistent interference and label noise; Section S18, Tables S19 and S20: Application to CL-Drive.

## Abbreviations

The following abbreviations are used in this manuscript:

AUC	Area under the curve
CI	Confidence interval
ECG	Electrocardiography
EDA	Electrodermal activity
EEG	Electroencephalography
LOSO	Leave-one-participant-out
LR	Logistic regression
MTEP	Matched temporal evaluation protocol
PR	Precision–recall
RF	Random forest
ROC	Receiver operating characteristic
SD	Standard deviation
SVM	Support vector machine
