# Supplementary material for: MTEP: A Matched Temporal Evaluation Protocol for Cognitive Fatigue Detection from In-Task Self-Reports
Source: Diagnostics (Basel). 2026 Oct 28;16(21):3556. doi: 10.3390/diagnostics16213556 (PMC13762228; doi:10.3390/diagnostics16213556)
Supplement: Supplementary file 1 [file diagnostics-16-03556-s001.zip › diagnostics-4642868-supplementary.pdf]

Supplementary Materials

MTEP: A Matched Temporal Evaluation Protocol for Cognitive Fatigue Detection from In-Task Self-Reports

Every table in this document is generated from the files in results/ of the code release, by code/make_tables.py (Tables S1–S8; TABLES.md contains the same tables as plain text) and code/make_sim_tables.py (Table S9); Tables S10 to S20 are produced by the scripts named in Sections S11 and S13 to S18. Section references are to the main manuscript.

S1. Per-participant results under MTEP

Fold-wise ROC-AUC for each held-out participant in the controlled design at lead L = 1 (Section 3.6), identified by CogBeacon participant identifier as used in the corpus folder names. Rows correspond to the points in Figure S1. Five of the 20 participant identifiers never pressed the button and cannot contribute cases.

Table S1 *Per-participant ROC-AUC in the controlled design (L = 1), by CogBeacon participant identifier. Cases: number of case windows for that participant; folds with one or two cases can only take a few discrete AUC values, which is why pooled estimates are primary.*

Participant	Cases	Windows	Position	EEG	Facial	EEG+fac.	Task	All RF	All Tr.
0	9	36	0.519	0.370	0.621	0.551	0.646	0.638	0.564
1	6	24	0.546	0.472	0.565	0.537	0.741	0.602	0.426
2	6	24	0.519	0.454	0.421	0.481	0.741	0.574	0.630
3	11	44	0.463	0.537	0.758	0.730	0.534	0.612	0.590
4	5	20	0.613	0.600	0.560	0.773	0.693	0.827	0.560
6	5	20	0.480	0.773	0.840	0.667	0.680	0.653	0.333
9	4	16	0.562	0.542	0.646	0.833	0.688	0.646	0.729
10	1	4	0.667	0.333	0.333	0.000	1.000	1.000	1.000
11	4	16	0.521	0.646	0.438	0.625	0.396	0.458	0.479
12	2	8	0.667	0.417	1.000	0.500	0.750	0.583	0.667
13	2	8	0.333	0.583	0.917	0.917	1.000	1.000	1.000
14	3	12	0.556	0.481	0.407	0.519	1.000	1.000	0.370
16	2	8	0.583	0.417	0.417	0.500	1.000	1.000	1.000
17	1	4	0.333	0.333	1.000	0.333	1.000	1.000	1.000
19	7	28	0.503	0.422	0.735	0.571	0.776	0.789	0.918

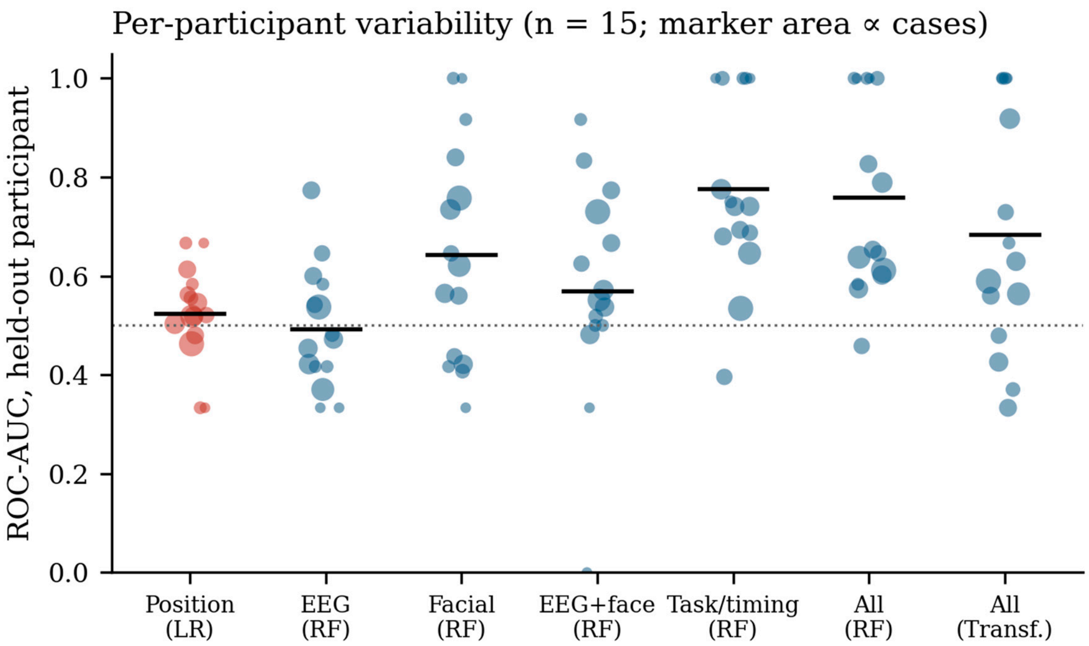

Figure S1 *ROC-AUC for each held-out participant in the controlled design at L = 1. Marker area is proportional to the participant's number of cases; horizontal bars mark unweighted means. Values are those of Table S1.*

Figure S2 shows the construction of the matched design and its pooled out-of-fold curves (Section 3.6).

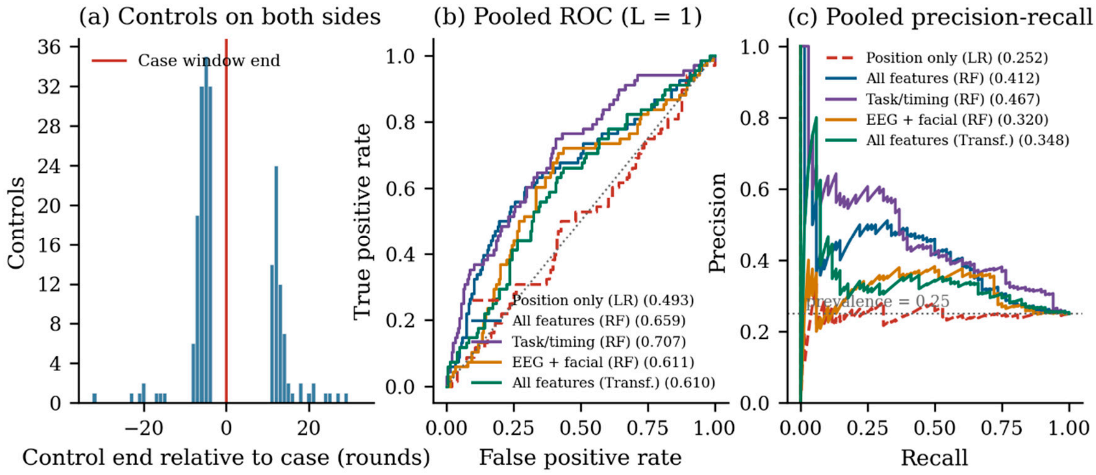

Figure S2 *The matched design at L = 1: (a) control positions, (b) ROC, (c) precision-recall. (a) Control window ends relative to their case, in rounds; controls fall on both sides, which is what the position certificate tests. (b, c) Pooled out-of-fold curves; the position-only model lies on the diagonal in (b) and at prevalence in (c). Parenthesised values are pooled ROC-AUC in (b) and pooled PR-AUC in (c); the dotted line in (c) marks class prevalence.*

S2. Complete sensitivity grid

All 33 design configurations at lead L = 1 (Section 3.9): 27 combinations of window length, minimum gap and maximum offset with three controls per case and seed 1337, plus three control ratios and three further seeds at the default configuration. The grid does not vary the lead (Table 7 of the main text) or the feature sets. The position

column is the position check, and Check marks whether its 95% CI includes 0.5; configurations marked FAIL are not interpreted (Section 3.9). A value below 0.5 means that position ranks controls slightly above cases.

Table S2 *Complete sensitivity grid (33 configurations) at lead L = 1: pooled ROC-AUC. W window length, g minimum gap to any press, m maximum offset, k controls per case.*

W	g	m	k	Seed	Cases	n	Position	Check	EEG+facial	Task	All
5	3	20	3	1337	74	296	0.508	FAIL	0.575	0.633	0.629
5	3	30	3	1337	74	296	0.508	FAIL	0.575	0.633	0.629
5	3	50	3	1337	74	296	0.508	FAIL	0.575	0.633	0.629
5	5	20	3	1337	74	296	0.526	FAIL	0.559	0.696	0.653
5	5	30	3	1337	74	296	0.526	FAIL	0.559	0.696	0.653
5	5	50	3	1337	74	296	0.526	FAIL	0.559	0.696	0.653
5	10	20	3	1337	74	296	0.536	FAIL	0.622	0.691	0.675
5	10	30	3	1337	74	296	0.536	FAIL	0.622	0.691	0.675
5	10	50	3	1337	74	296	0.536	FAIL	0.622	0.691	0.675
10	3	20	3	1337	68	272	0.505	pass	0.592	0.716	0.662
10	3	30	3	1337	68	272	0.505	pass	0.592	0.716	0.662
10	3	50	3	1337	68	272	0.505	pass	0.592	0.716	0.662
10	5	20	3	1337	68	272	0.493	pass	0.611	0.707	0.659
10	5	30	1	1337	68	136	0.472	FAIL	0.521	0.699	0.639
10	5	30	2	1337	68	204	0.514	pass	0.575	0.726	0.676
10	5	30	3	7	68	272	0.499	pass	0.580	0.739	0.678
10	5	30	3	42	68	272	0.502	pass	0.619	0.725	0.703
10	5	30	3	1337	68	272	0.493	pass	0.611	0.707	0.659
10	5	30	3	2024	68	272	0.500	pass	0.612	0.706	0.677
10	5	30	5	1337	68	408	0.493	pass	0.579	0.708	0.657
10	5	50	3	1337	68	272	0.493	pass	0.611	0.707	0.659
10	10	20	3	1337	68	272	0.531	FAIL	0.580	0.726	0.698
10	10	30	3	1337	68	272	0.531	FAIL	0.580	0.726	0.698
10	10	50	3	1337	68	272	0.531	FAIL	0.580	0.726	0.698
15	3	20	3	1337	60	240	0.507	pass	0.573	0.656	0.624
15	3	30	3	1337	60	240	0.507	pass	0.573	0.656	0.624
15	3	50	3	1337	60	240	0.507	pass	0.573	0.656	0.624
15	5	20	3	1337	60	240	0.500	pass	0.494	0.695	0.616
15	5	30	3	1337	60	240	0.500	pass	0.494	0.695	0.616
15	5	50	3	1337	60	240	0.500	pass	0.494	0.695	0.616
15	10	20	3	1337	58	231	0.444	FAIL	0.515	0.682	0.617
15	10	30	3	1337	58	231	0.444	FAIL	0.515	0.682	0.617
15	10	50	3	1337	58	231	0.444	FAIL	0.515	0.682	0.617

S3. Complete paired significance tests

All paired comparisons of Section 3.8, with the Wilcoxon statistic, the test variant used, raw and Holm-corrected p-values, and participant-level wins, ties and losses.

Table S3 *Complete paired tests. W: Wilcoxon statistic; test: exact null when no zero or tied differences, otherwise normal approximation; zero differences discarded (Wilcoxon zero method).*

A	B	n	W/T/L	Median diff.	W	Test	p	p (Holm)	Effect
All features (RF)	All features (Transformer)	15	6/4/5	+0.000	22.0	normal approx.	.328	1.000	+0.33
All features (RF)	All features (LR)	15	8/3/4	+0.019	21.0	normal approx.	.158	.948	+0.46

A	B	n	W/T/L	Median diff.	W	Test	p	p (Holm)	Effect
All features (RF)	EEG + facial (RF)	15	11/0/4	+0.083	28.0	exact	.073	.511	+0.53
EEG + facial (RF)	EEG only (RF)	15	11/1/3	+0.083	21.0	normal approx.	.048	.383	+0.60
EEG + facial (RF)	Facial only (RF)	15	6/1/8	-0.028	44.0	normal approx.	.594	1.000	-0.16
All features (RF)	Task/timing only (RF)	15	4/5/6	+0.000	20.0	normal approx.	.445	1.000	-0.27
All features (RF)	Position only (LR)	15	13/0/2	+0.173	7.5	normal approx.	.003	.026	+0.88
EEG + facial (RF)	Position only (LR)	15	8/1/6	+0.033	37.0	normal approx.	.331	1.000	+0.30
Task/timing only (RF)	Position only (LR)	15	14/0/1	+0.200	5.0	normal approx.	.002	.018	+0.92
All features (Transformer)	All features (LR)	15	5/4/6	+0.000	27.5	normal approx.	.625	1.000	-0.17

S4. Feature inventory

All 97 per-round features in the column order of the released feature matrix (Section 2.2.3), with their group and the fraction of rounds on which each was missing before imputation.

Table S4 *Feature inventory in the column order of data/dataset.npz, with missingness before imputation (training-fold median). Electrode labels follow the Muse SDK channel order (TP9, AF7, AF8, TP10); the corpus documentation does not list the order. Tags: A-prefixed = absolute band power, unprefixed = relative band power (a alpha, b beta, d delta, g gamma, t theta), as documented in the corpus README.*

#	Feature	Group	Description	Missing
1	eeg_Aa_mean_ch0	EEG	electrode TP9	0.4%
2	eeg_Aa_mean_ch1	EEG	electrode AF7	0.4%
3	eeg_Aa_mean_ch2	EEG	electrode AF8	0.4%
4	eeg_Aa_mean_ch3	EEG	electrode TP10	0.4%
5	eeg_Ab_mean_ch0	EEG	electrode TP9	0.4%
6	eeg_Ab_mean_ch1	EEG	electrode AF7	0.4%
7	eeg_Ab_mean_ch2	EEG	electrode AF8	0.4%
8	eeg_Ab_mean_ch3	EEG	electrode TP10	0.4%
9	eeg_Ad_mean_ch0	EEG	electrode TP9	0.4%
10	eeg_Ad_mean_ch1	EEG	electrode AF7	0.4%
11	eeg_Ad_mean_ch2	EEG	electrode AF8	0.4%
12	eeg_Ad_mean_ch3	EEG	electrode TP10	0.4%
13	eeg_Ag_mean_ch0	EEG	electrode TP9	0.4%
14	eeg_Ag_mean_ch1	EEG	electrode AF7	0.4%
15	eeg_Ag_mean_ch2	EEG	electrode AF8	0.4%
16	eeg_Ag_mean_ch3	EEG	electrode TP10	0.4%
17	eeg_At_mean_ch0	EEG	electrode TP9	0.4%
18	eeg_At_mean_ch1	EEG	electrode AF7	0.4%
19	eeg_At_mean_ch2	EEG	electrode AF8	0.4%
20	eeg_At_mean_ch3	EEG	electrode TP10	0.4%
21	eeg_a_mean_ch0	EEG	electrode TP9	1.3%
22	eeg_a_mean_ch1	EEG	electrode AF7	2.9%
23	eeg_a_mean_ch2	EEG	electrode AF8	32.8%
24	eeg_a_mean_ch3	EEG	electrode TP10	1.1%
25	eeg_b_mean_ch0	EEG	electrode TP9	1.3%
26	eeg_b_mean_ch1	EEG	electrode AF7	2.9%
27	eeg_b_mean_ch2	EEG	electrode AF8	32.8%
28	eeg_b_mean_ch3	EEG	electrode TP10	1.1%
29	eeg_d_mean_ch0	EEG	electrode TP9	1.3%
30	eeg_d_mean_ch1	EEG	electrode AF7	2.9%
31	eeg_d_mean_ch2	EEG	electrode AF8	32.8%

#	Feature	Group	Description	Missing
32	eeg_d_mean_ch3	EEG	electrode TP10	1.1%
33	eeg_g_mean_ch0	EEG	electrode TP9	1.3%
34	eeg_g_mean_ch1	EEG	electrode AF7	2.9%
35	eeg_g_mean_ch2	EEG	electrode AF8	32.8%
36	eeg_g_mean_ch3	EEG	electrode TP10	1.1%
37	eeg_t_mean_ch0	EEG	electrode TP9	1.3%
38	eeg_t_mean_ch1	EEG	electrode AF7	2.9%
39	eeg_t_mean_ch2	EEG	electrode AF8	32.8%
40	eeg_t_mean_ch3	EEG	electrode TP10	1.1%
41	eeg_a_sd_ch0	EEG	electrode TP9	1.3%
42	eeg_a_sd_ch1	EEG	electrode AF7	2.9%
43	eeg_a_sd_ch2	EEG	electrode AF8	32.8%
44	eeg_a_sd_ch3	EEG	electrode TP10	1.1%
45	eeg_b_sd_ch0	EEG	electrode TP9	1.3%
46	eeg_b_sd_ch1	EEG	electrode AF7	2.9%
47	eeg_b_sd_ch2	EEG	electrode AF8	32.8%
48	eeg_b_sd_ch3	EEG	electrode TP10	1.1%
49	eeg_d_sd_ch0	EEG	electrode TP9	1.3%
50	eeg_d_sd_ch1	EEG	electrode AF7	2.9%
51	eeg_d_sd_ch2	EEG	electrode AF8	32.8%
52	eeg_d_sd_ch3	EEG	electrode TP10	1.1%
53	eeg_g_sd_ch0	EEG	electrode TP9	1.3%
54	eeg_g_sd_ch1	EEG	electrode AF7	2.9%
55	eeg_g_sd_ch2	EEG	electrode AF8	32.8%
56	eeg_g_sd_ch3	EEG	electrode TP10	1.1%
57	eeg_t_sd_ch0	EEG	electrode TP9	1.3%
58	eeg_t_sd_ch1	EEG	electrode AF7	2.9%
59	eeg_t_sd_ch2	EEG	electrode AF8	32.8%
60	eeg_t_sd_ch3	EEG	electrode TP10	1.1%
61	eeg_theta_alpha_ch0	EEG	electrode TP9	1.3%
62	eeg_theta_alpha_ch1	EEG	electrode AF7	2.9%
63	eeg_theta_alpha_ch2	EEG	electrode AF8	32.8%
64	eeg_theta_alpha_ch3	EEG	electrode TP10	1.1%
65	eeg_engagement_ch0	EEG	electrode TP9	1.3%
66	eeg_engagement_ch1	EEG	electrode AF7	2.9%
67	eeg_engagement_ch2	EEG	electrode AF8	32.8%
68	eeg_engagement_ch3	EEG	electrode TP10	1.1%
69	eeg_quality_ch0	EEG	electrode TP9	0.4%
70	eeg_quality_ch1	EEG	electrode AF7	0.4%
71	eeg_quality_ch2	EEG	electrode AF8	0.4%
72	eeg_quality_ch3	EEG	electrode TP10	0.4%
73	face_eye_extent_mean	Facial	eye landmark extent ratio (h/w), mean	11.2%
74	face_eye_extent_sd	Facial	eye extent ratio, SD	11.2%
75	face_mouth_extent_mean	Facial	mouth extent ratio, mean	11.2%
76	face_mouth_extent_sd	Facial	mouth extent ratio, SD	11.2%
77	face_bbox_w_px	Facial	face box width, pixels	0.0%
78	face_bbox_h_px	Facial	face box height, pixels	0.0%
79	face_bbox_x_px	Facial	face box x, pixels	0.0%
80	face_bbox_y_px	Facial	face box y, pixels	0.0%
81	face_motion_mean	Facial	centroid motion / box width, mean	0.0%

#	Feature	Group	Description	Missing
82	face_motion_sd	Facial	centroid motion, SD	0.0%
83	face_motion_max	Facial	centroid motion, max	0.0%
84	perf_round_frames	Task/timing	video frames in round (duration at 2 fps)	0.0%
85	face_head_x_sd	Facial	centroid x / box width, SD	0.0%
86	face_head_y_sd	Facial	centroid y / box width, SD	0.0%
87	perf_level	Task/timing		0.0%
88	perf_score	Task/timing		0.0%
89	perf_response_time	Task/timing		0.0%
90	perf_correct	Task/timing		0.0%
91	perf_question	Task/timing		0.0%
92	perf_persistence	Task/timing		0.0%
93	perf_round_norm	Task/timing	round index / session length	0.0%
94	perf_d_nonper_err	Task/timing		0.0%
95	perf_d_per_err	Task/timing		0.0%
96	perf_rt_dev	Task/timing	response time z-score vs last 10 rounds	0.0%
97	perf_acc_run	Task/timing	accuracy over last 10 rounds	0.0%

S5. Position-decile matching on the cumulative-state label

Supporting Section 3.4. Positive and negative windows of the state label were balanced within session-position deciles, either across the whole corpus or within each participant, and the models were re-evaluated by grouped leave-one-participant-out cross-validation.

Table S5 Failed attempts to remove the position confound from the cumulative-state label by position-decile matching (Section 3.4). The position-only model remains far above chance under both.

Matching	Model	Windows	Pooled ROC-AUC [95% CI]	Fold ROC-AUC
Corpus-level decile matching	Position only (LR)	4328	0.703 [0.628, 0.798]	0.923 ± 0.096
Corpus-level decile matching	All features (LR)	4328	0.704 [0.608, 0.791]	0.790 ± 0.096
Within-participant decile matching	Position only (LR)	2278	0.894 [0.848, 0.938]	0.919 ± 0.093
Within-participant decile matching	All features (LR)	2278	0.655 [0.592, 0.726]	0.733 ± 0.139

S6. Model settings

Table S6 *Model settings, identical in both studies. In every outer fold all models are trained on the same participants; the validation participants are used only for Transformer early stopping.*

Model	Settings
Logistic regression	L2, C = 1.0, class_weight = balanced, max_iter = 3000; input: window mean, SD and final value
Random forest	400 trees, min_samples_leaf = 3, class_weight = balanced_subsample; same input
SVM (Study 1)	RBF kernel, C = 1.0, gamma = scale, class_weight = balanced; score = logistic(decision function), so 0.5 is the SVM decision boundary; same input
Transformer	linear projection to 48, sinusoidal positions, 2 layers, 4 heads, GELU, dropout 0.2, AdamW (lr 1e-3, wd 1e-2), batch 256, ≤ 15 epochs, patience 4 on validation ROC-AUC, class-weighted BCE, gradient clipping 1.0
Validation split	1/8 of training participants (at least one), drawn once per outer fold with seed 1337
Hyperparameter tuning	none for any model

Model	Settings
Early-stopping fallback folds	state|all|transformer: 2; state|eeg_only|transformer: 2; state|face_only|transformer: 2; state|task_only|transformer: 2; state|eeg+face|transformer: 2; event_d0|all|transformer: 2; event_d1|all|transformer: 2; event_d3|all|transformer: 2; event_d5|all|transformer: 2; All features (Transformer): 0

S7. Case-control construction and the press round

At the default configuration (W = 10, g = 5, m = 30, k = 3, L = 1) the 79 presses yielded 68 cases: 2 presses fell within the first W rounds of their session and 9 were discarded because the case window, ending one round before the press, contained an earlier press. Every case received its full complement of three controls; no control window is used by more than one case; 34% of controls lie after their case and the mean absolute offset is 9.3 rounds. The per-window offsets, labels, participant identifiers and matched-set identifiers are stored in results/study2/design.json. Table S7 compares rounds that contain a press with all other rounds on three features (Section 3.5).

Table S7 Medians of selected per-round features in the 79 rounds containing a press and in all other rounds (Section 3.5).

Feature	Press rounds	Other rounds	Ratio
Maximum centroid displacement (box widths)	0.607	0.0304	19.94
Video frames in round	13	9	1.44
Response time	3	2	1.50

S8. Study 1: all configurations

All 14 configurations of the conventional sliding-window protocol (Section 3.4), including the single-modality ablations under the state label and the event target at horizons D = 0, 1, 3 and 5 (Figure 4a of the main text). The main text reports the key values in Section 3.4.

Table S8 *Study 1, conventional sliding-window protocol under grouped leave-one-participant-out, all configurations. Pooled ROC-AUC with participant-clustered bootstrap 95% CI; fold-wise values mean ± SD over participants with both classes (Folds).*

Configuration	Prev.	Pooled ROC-AUC [95% CI]	Pooled PR-AUC	Fold ROC-AUC	Fold bal. acc.	Folds
State: all features (Transformer)	0.346	0.678 [0.622, 0.744]	0.590	0.850 ± 0.109	0.694 ± 0.107	15/20
State: all features (LR)	0.346	0.767 [0.701, 0.828]	0.630	0.868 ± 0.087	0.724 ± 0.100	15/20
State: all features (RF)	0.346	0.732 [0.668, 0.802]	0.531	0.893 ± 0.032	0.717 ± 0.120	15/20
State: all features (SVM)	0.346	0.750 [0.682, 0.815]	0.600	0.878 ± 0.065	0.723 ± 0.087	15/20
State: position only (normalised) (LR)	0.346	0.789 [0.734, 0.852]	0.601	0.933 ± 0.054	0.831 ± 0.047	15/20
State: elapsed rounds only (LR)	0.346	0.774 [0.724, 0.832]	0.597	0.915 ± 0.067	0.819 ± 0.060	15/20
State: EEG only (Transformer)	0.346	0.563 [0.442, 0.692]	0.452	0.603 ± 0.194	0.555 ± 0.109	15/20
State: facial only (Transformer)	0.346	0.545 [0.426, 0.655]	0.441	0.518 ± 0.191	0.492 ± 0.101	15/20
State: task/timing only (Transformer)	0.346	0.721 [0.669, 0.777]	0.532	0.819 ± 0.106	0.733 ± 0.084	15/20
State: EEG + facial (Transformer)	0.346	0.538 [0.419, 0.660]	0.412	0.564 ± 0.181	0.526 ± 0.095	15/20
Event, D = 0: all features (Transformer)	0.012	0.708 [0.582, 0.830]	0.051	0.872 ± 0.115	0.733 ± 0.165	15/20

Configuration	Prev.	Pooled ROC-AUC [95% CI]	Pooled PR-AUC	Fold ROC-AUC	Fold bal. acc.	Folds
Event, D = 1: all features (Transformer)	0.025	0.728 [0.650, 0.815]	0.116	0.804 ± 0.139	0.709 ± 0.112	15/20
Event, D = 3: all features (Transformer)	0.049	0.607 [0.529, 0.715]	0.098	0.748 ± 0.155	0.624 ± 0.111	15/20
Event, D = 5: all features (Transformer)	0.072	0.614 [0.521, 0.711]	0.140	0.704 ± 0.146	0.582 ± 0.068	15/20

S9. The certified lead under other estimators and window lengths

The displacement sweep of Section 3.5 repeated on all features with each of the three classical estimators and with the random forest at window lengths of 5, 10 and 15 rounds (Section 3.9). All six runs certify at a lead of one round.

Table S9 The certified lead under other estimators and other window lengths, on all features.

Varied	Setting	L = 0	L = 1	L = 2	L = 3	D(0 to 1)	D(1 to 2)	D(2 to 3)	Certified lead
Estimator (W = 10)	Random forest	0.888	0.659	0.645	0.590	+0.228	+0.014	+0.055	L = 1
Estimator (W = 10)	Logistic regression	0.823	0.602	0.601	0.590	+0.221	+0.001	+0.011	L = 1
Estimator (W = 10)	SVM	0.859	0.664	0.638	0.602	+0.194	+0.026	+0.036	L = 1
Window length (random forest)	W = 5 rounds	0.882	0.653	0.610	0.591	+0.229	+0.043	+0.019	L = 1
Window length (random forest)	W = 10 rounds	0.888	0.659	0.645	0.590	+0.228	+0.014	+0.055	L = 1
Window length (random forest)	W = 15 rounds	0.874	0.616	0.640	0.584	+0.258	-0.024	+0.056	L = 1

Each row repeats the displacement sweep and applies the magnitude rule with a threshold of 0.10. AUC values are pooled out-of-fold ROC-AUC at leads of 0 to 3 rounds, with g, m and k at their defaults throughout. The estimator rows share the folds and windows of the main analysis; the window-length rows rebuild the design at each W, which changes the number of cases available before the lead is applied from 78 at W = 5 to 74 at W = 15.

S10. Calibration of the certificates: implementation details

These details complete Section 2.5 of the main text. Replacing the feature vectors of press rounds was rejected as a reference condition because pilot runs confirmed that such a repair introduces a discontinuity which the certificate itself detects; the synthetic-event construction was used instead, and it placed a mean of 66.1 of the 79 events per replicate.

The latent magnitude $z = a(1 + e)$, with e standard normal, has an event-to-event scatter equal to its mean, as an act whose vigour varies between occurrences would. Six of the 97 features are piecewise constant within a window, so that an offset on one of them would be visible at any amplitude however small; these are not eligible for injection, leaving 91. Injected offsets are snapped to each feature's recording resolution, since several features are counts.

Noise draws are held fixed across amplitudes within a replicate, so the dose-response is paired. Because no L = 1 window can contain an event round in the spike condition, the maximum absolute difference between the L = 1 feature tensors at amplitude 0 and at amplitude 50 was exactly zero in all 20 replicates, confirming that injection at the event round cannot reach the design read at a lead of one round.

S11. Calibration of the report-independence certificate by corpus size

The calibration of Section 2.5 repeated on random subsets of 12, 9 and 6 of the 15 participants who pressed, and on all 15 as an independent second set of replicates at the full size (Section 3.2). In each replicate the matched design is rebuilt around synthetic events, the real presses are blocked, and a component of known amplitude, in local standard deviations of the full corpus, is injected on the event round (act) or as a smooth bump around it (state, amplitudes 0.5 and 1.0). Decisions use the magnitude rule at the adopted threshold of 0.10. Produced by code/run_samplesize.py.

Table S10 Calibration of the report-independence certificate by corpus size, 20 replicates per size.

Participants	Replicates	Mean events	Reference certified	Genuine state certified	Act 0.05 refused	Act 0.1 refused	Threshold for 95% specificity	Largest sound statistic
15	20	66.0	20/20	40/40	13/20	15/20	0.066	+0.076
12	20	53.4	20/20	40/40	11/20	14/20	0.080	+0.096
9	20	39.8	19/20	40/40	10/20	12/20	0.074	+0.133
6	20	25.6	19/20	38/40	2/20	3/20	0.095	+0.113

Mean events: case windows at lead 1. The threshold for 95% specificity is the 95th percentile of the displacement statistic over the sound designs (reference and genuine state) at that size, the threshold a corpus of that size would calibrate; every value lies below the adopted 0.10.

S12. Contents of the code and data release

The release contains the dataset builder with its label-feature separation and name check; the Study 1 and Study 2 harnesses; the lead sweep and the displacement statistic with its bootstrap interval, which together implement the report-independence certificate; the calibration of both certificates, comprising the synthetic-event construction that supplies a report-independent reference design, the injection of act-shaped and state-shaped components of known amplitude, and the position-imbalance sweep; the position-decile matching attempts; the sensitivity grid; the matched-set permutation null; the paired tests; the evaluation of the certified detector on the full recording stream; the calibration of the certificate by corpus size; the reproduction of the published CogBeacon protocols; the derived feature matrix; for every configuration of both studies, the out-of-fold prediction for every window together with its label and participant; one file per calibration replicate; and the scripts that generate every table and figure. A single script, run_all.sh, runs these steps in order from the raw corpus, skips steps that have already completed so that an interrupted run can be restarted, and recomputes everything with --force. Establishing the two certificates first is recommended: the position-only model must fall to chance on the matched sample, and the displacement statistic identifies the lead at which the estimate should be read. The calibration is the part of the release that says what a certificate is worth, and it can be re-run on a new corpus to obtain specificity and sensitivity for that corpus rather than borrowing the figures reported here.

The CogBeacon corpus itself is not redistributed. It is available from its authors, and the release documents the expected directory layout. CL-Drive is likewise not redistributed; it is available at https://doi.org/10.5683/SP3/JJ2YZZ.

S13. Revision analyses: preparation before the press and the fixed-cohort lead sweep

All analyses in Sections S13 to S18 were added in revision and use the released feature matrix, the folds, estimators and settings of Study 2, and scikit-learn 1.9.1; they are produced by code/run_revision.py, code/run_sim_extended.py and the revised code/run_headtohead.py.

Table S11 Medians of act-sensitive features in the press round and the three rounds before it, against rounds more than five rounds from any press (Section 3.5).

Feature	Other rounds	Press − 3	Press − 2	Press − 1	Press round
Maximum landmark-centroid displacement	0.0301	0.0179 (0.59×)	0.0334 (1.11×)	0.117 (3.87×)	0.607 (20.15×)
Mean landmark-centroid displacement	0.0124	0.00792 (0.64×)	0.00978 (0.79×)	0.0413 (3.34×)	0.197 (15.96×)
Round duration (frames)	9	9 (1.00×)	10 (1.11×)	10 (1.11×)	13 (1.44×)
Response time	2	2 (1.00×)	2 (1.00×)	2 (1.00×)	3 (1.50×)

Ratios to the median in other rounds in brackets. Rounds: 79 press rounds, 79 rounds before them, 6167 other rounds; a round that is itself a press round is not counted as a preceding round.

Table S12 The lead sweep on a fixed cohort: 63 cases with the same 189 controls at every lead (15 participants).

Feature set	L = 0	L = 1	L = 2	L = 3	D(0 to 1)	D(1 to 2)	D(2 to 3)	Lead
All features	0.895	0.690	0.657	0.598	+0.205 [+0.112, +0.284]	+0.033 [−0.033, +0.094]	+0.059 [+0.006, +0.129]	L* = 1; reported at L = 1
EEG + facial	0.806	0.596	0.539	0.528	+0.210 [+0.149, +0.299]	+0.057 [−0.007, +0.125]	+0.011 [−0.052, +0.055]	L* = 1; reported at L = 1
EEG only	0.524	0.498	0.534	0.522	+0.026 [−0.042, +0.085]	−0.037 [−0.096, +0.028]	+0.012 [−0.039, +0.071]	L* = 0; reported at L = 1
Facial only	0.826	0.614	0.452	0.449	+0.212 [+0.142, +0.284]	+0.162 [+0.104, +0.218]	+0.003 [−0.073, +0.078]	L* = 2; reported at L = 2
Task/timing only	0.824	0.730	0.665	0.627	+0.094 [+0.029, +0.149]	+0.066 [+0.018, +0.120]	+0.038 [−0.013, +0.102]	L* = 0; reported at L = 1
Position only (LR)	0.517	0.499	0.509	0.517	—	—	—	—

Cases are the presses whose case windows are admissible at every lead from 0 to 3; each keeps the same three controls, drawn by the design's balancing rule relative to the lead-1 case window, so only the case window moves. Steps carry participant-clustered bootstrap 95% intervals. L* is the smallest lead beyond which every further step is below 0.10; estimates are reported at max(1, L*).

S14. What the task signal is

Table S13 Decomposition of the task signal: random forest, pooled ROC-AUC [participant-clustered 95% CI] at leads 1 and 2.

Feature set	L = 1	L = 2
All features	0.661 [0.616, 0.733]	0.648 [0.611, 0.703]
All features without position-like variables	0.662 [0.622, 0.727]	0.637 [0.584, 0.698]
Task and timing	0.704 [0.641, 0.788]	0.684 [0.635, 0.747]
Task and timing without position-like variables	0.701 [0.642, 0.787]	0.681 [0.625, 0.749]
… and without difficulty level	0.697 [0.633, 0.782]	0.682 [0.626, 0.752]
Performance variables only (8)	0.695 [0.638, 0.782]	0.696 [0.638, 0.768]
Round duration only	0.546 [0.456, 0.622]	0.515 [0.423, 0.605]

Position-like variables: normalised round position and question index. Performance variables: score, response time, correctness, perseveration flag, increments in perseverative and non-perseverative errors, response-time deviation and ten-round running accuracy.

Table S14 Cases against earlier and against later controls in the matched design at L = 1, from the stored out-of-fold scores of Table 8.

Model	All controls	Earlier controls	Later controls
All features (RF)	0.659 [0.612, 0.733]	0.619 [0.551, 0.712]	0.737 [0.685, 0.805]
Task and timing (RF)	0.707 [0.645, 0.789]	0.651 [0.579, 0.752]	0.813 [0.719, 0.902]
EEG + facial (RF)	0.611 [0.551, 0.672]	0.606 [0.536, 0.675]	0.620 [0.543, 0.694]
Position only (LR)	0.493 [0.482, 0.512]	0.502 [0.472, 0.540]	0.476 [0.430, 0.522]

Each column scores the 68 cases against the controls on one side of their case only. Time-on-task drift would make later controls resemble cases; a post-report effect can reach only later controls. Window overlap in the design: 61% of controls overlap their case window, sharing 4.5 rounds on average (median absolute offset 7 rounds).

S15. Control-side variants

Table S15 Control-side variants of the matched design at L = 1.

Controls	Cases/controls	Mean offset	Position (LR)	Position family (RF)	P3	All (RF)	Task (RF)	EEG + facial (RF)
Bilateral (design)	68/204	+0.32	0.493 [0.481, 0.512]	0.554 [0.490, 0.625]	granted	0.661 [0.616, 0.733]	0.704 [0.641, 0.788]	0.610 [0.549, 0.672]
Earlier only	65/195	-7.19	0.583 [0.577, 0.603]	0.581 [0.531, 0.634]	refused	0.630 [0.558, 0.723]	0.696 [0.627, 0.787]	0.550 [0.503, 0.591]
Later only	58/173	+15.42	0.653 [0.628, 0.680]	0.635 [0.529, 0.744]	refused	0.718 [0.622, 0.829]	0.818 [0.721, 0.920]	0.451 [0.380, 0.513]
Bilateral, no overlap	68/199	-2.78	0.545 [0.516, 0.589]	0.588 [0.533, 0.653]	refused	0.708 [0.635, 0.791]	0.735 [0.652, 0.838]	0.588 [0.531, 0.655]

Earlier only and later only draw each control at random from the three nearest eligible windows on one side of the case; bilateral, no overlap applies the design's balancing rule to candidates whose windows share no round with the case window or with each other. Sets without any control are dropped. P3 column: granted when the intervals of the linear, forest and position-family models computed for this table all include 0.5; the one-sided variants already fail on the linear model. Pooled ROC-AUC with participant-clustered 95% CI.

Paired by participant against the bilateral design (fold-wise ROC-AUC, Wilcoxon signed-rank): before all wins/ties/losses 2/2/11, $p = 0.032$; before task wins/ties/losses 6/3/6, $p = 0.970$; after all wins/ties/losses 7/5/3, $p = 0.193$; after task wins/ties/losses 9/5/1, $p = 0.014$; nonoverlap all wins/ties/losses 7/4/4, $p = 0.620$; nonoverlap task wins/ties/losses 6/4/5, $p = 0.638$.

S16. Nonlinear position baselines and the power of the position certificate

Table S16 Position-only baselines on the matched design at L = 1.

Model	Pooled ROC-AUC [95% CI]	Interval includes 0.5
Position, logistic regression	0.493 [0.481, 0.512]	yes
Position, cubic spline + LR	0.489 [0.446, 0.538]	yes
Position, random forest	0.533 [0.430, 0.625]	yes
Position, gradient boosting	0.507 [0.395, 0.600]	yes
Position, 25-nearest neighbours	0.533 [0.457, 0.603]	yes
Position family, random forest	0.554 [0.490, 0.625]	yes
Position family, gradient boosting	0.557 [0.485, 0.637]	yes

Each model receives the window summary (mean, SD and final value) of its inputs. Position family: normalised round position, question index, elapsed rounds and session length.

Table S17 Power of the position certificate against a dispersion imbalance that leaves the mean offset balanced.

γ far	Mean offset	Mean |offset|	LR	Spline LR	RF	GBM	kNN	Family RF	Revised rule
0.00	+0.34	9.2	0/10	0/10	0/10	0/10	0/10	1/10	0/10
0.25	-0.83	18.7	2/10	0/10	2/10	0/10	0/10	3/10	4/10
0.50	-1.10	29.3	0/10	10/10	6/10	5/10	8/10	7/10	10/10
0.75	-1.91	37.1	0/10	10/10	10/10	10/10	10/10	10/10	10/10
1.00	-2.90	43.9	0/10	10/10	10/10	10/10	10/10	10/10	10/10

With probability γ far each control is the farthest remaining eligible window on the side chosen by the balancing rule instead of one of the three nearest, so controls stay balanced before and after their case but spread away from it: a nonlinear dependence of the label on position that a linear model cannot represent. Cells give the number of replicates in which the model's interval excluded 0.5 (imbalance detected). Revised rule: refused when any of the five models on the position variable (LR, spline LR, RF, GBM, kNN) detects imbalance.

S17. The report-independence certificate under other temporal profiles, persistent interference and label noise

The calibration of Section 2.5 extended to departures from its two shapes, on synthetic events placed away from every real press, with new seeds. Act-borne conditions carry no state, so a sound design must not conclude that a detector works once it is read at the reported lead; state conditions carry a genuine state with a non-Gaussian temporal profile or a noisy report, so a sound design must conclude that it does. Every condition is run at leads 0, 1 and 2, and at 3 for the preparation conditions, under the folds and estimator of Study 2. Produced by code/run_sim_extended.py.

Table S18 Extended calibration of the report-independence certificate, 10 replicates per condition.

Condition	Truth	AUC L = 0	AUC L = 1	AUC L = 2	D(0 to 1)	D(1 to 2)	L* (0/1/2/3)	Says yes	Correct
Nothing injected [0.0]	none	0.515 (0.042)	0.499 (0.033)	0.493 (0.048)	+0.016 (0.054)	+0.006 (0.056)	9/0/0/0 (+1 none)	0/10	10/10
Act + preparation (0.5 on preceding round) [0.1]	none	0.757 (0.089)	0.730 (0.087)	0.493 (0.048)	+0.027 (0.028)	+0.236 (0.103)	1/0/9/0	0/10	10/10
Preparation only (preceding round) [0.1]	none	0.698 (0.117)	0.734 (0.095)	0.493 (0.048)	-0.036 (0.073)	+0.240 (0.106)	1/0/9/0	0/10	10/10
Act, heavy-tailed magnitudes [0.05]	none	0.730 (0.111)	0.499 (0.033)	0.493 (0.048)	+0.231 (0.115)	+0.006 (0.056)	1/8/0/0 (+1 none)	0/10	10/10
Persistent interference, step [0.1]	none	0.700 (0.121)	0.496 (0.032)	0.488 (0.052)	+0.204 (0.112)	+0.009 (0.056)	2/8/0/0	0/10	10/10
Persistent interference, step [1.0]	none	0.778 (0.089)	0.516 (0.028)	0.517 (0.030)	+0.262 (0.098)	-0.001 (0.046)	1/9/0/0	1/10	9/10
Persistent interference, decay τ = 5 [1.0]	none	0.798 (0.064)	0.519 (0.032)	0.522 (0.038)	+0.279 (0.061)	-0.003 (0.051)	0/10/0/0	2/10	8/10
State, abrupt onset 3 rounds before [1.0]	state	0.796 (0.080)	0.785 (0.091)	0.774 (0.103)	+0.010 (0.028)	+0.011 (0.025)	10/0/0/0	10/10	10/10
State, linear ramp over 10 rounds [1.0]	state	0.623 (0.068)	0.602 (0.061)	0.576 (0.072)	+0.021 (0.039)	+0.026 (0.037)	10/0/0/0	9/10	9/10
State, exponential rise, fast decay [1.0]	state	0.746 (0.042)	0.696 (0.040)	0.631 (0.062)	+0.050 (0.021)	+0.065 (0.039)	9/0/0/0 (+1 none)	9/10	9/10
State, heavy-tailed magnitudes [1.0]	state	0.690 (0.066)	0.672 (0.075)	0.641 (0.092)	+0.018 (0.032)	+0.031 (0.048)	9/0/0/0 (+1 none)	9/10	9/10

Condition	Truth	AUC L = 0	AUC L = 1	AUC L = 2	D(0 to 1)	D(1 to 2)	L* (0/1/2/3)	Says yes	Correct
State, report jittered ±3 rounds [1.0]	state	0.686 (0.055)	0.661 (0.041)	0.641 (0.048)	+0.025 (0.031)	+0.020 (0.027)	10/0/0/0	10/10	10/10
State, as many unreported episodes [1.0]	state	0.690 (0.034)	0.671 (0.037)	0.671 (0.028)	+0.019 (0.017)	0.000 (0.034)	10/0/0/0	10/10	10/10
State, half of reports without state [1.0]	state	0.570 (0.046)	0.536 (0.041)	0.545 (0.048)	+0.035 (0.037)	-0.010 (0.038)	10/0/0/0	2/10	2/10

Each condition is injected at synthetic events placed away from every real press (Section 2.5) with the amplitude in brackets, in local standard deviations. AUC and steps: mean (SD) across replicates. L*: the smallest lead beyond which every further step is below 0.10, counted across replicates; the estimate is read at max(1, L*). Says yes: MTEP concludes that a detector works, i.e. the participant-clustered 95% interval of the pooled ROC-AUC at the reported lead excludes 0.5. Correct: says yes exactly when a state is present.

S18. Application to CL-Drive

CL-Drive [Angkan et al., IEEE Trans. Intell. Transp. Syst. 2024] records 21 drivers over nine induced complexity levels of 3 min each, with a spoken 1–9 cognitive-load rating every 10 s after an audio cue. EEG (Muse, TP9, AF7, AF8, TP10), EDA (Shimmer conductance) and gaze (Tobii pupil, blinks, velocity, fixation dispersion, validity, saccade amplitude) were used; ECG was not. The cue for the rating of unit k is at 120 + 10k s of each level recording, and the spoken report was located from power above 45 Hz at the temporal electrodes, which rose from 1.5 to 4.5 s after the cue in the average over all cues. Each unit is summarised over 3-s windows ending 4.5 s after the cue (the report itself), 1.0 s after, and 0.5 and 2.0 s before it (report-free), and 5.0 s before it (the previous report), and over the published 10-s segment; EEG features are log band powers (delta to gamma and 45–110 Hz) with theta/alpha and engagement ratios per electrode, EDA features the mean, SD and slope of conductance, and every feature is expressed relative to the level's own pre-drive baseline recording. Produced by code/cldrive/build_cldrive.py and code/cldrive/run_cldrive.py in the code release.

Table S19 CL-Drive under the published protocol: accuracy (%) for high (5–9) against low (1–4) rated load on 10-s segments.

Features	Published, 10-fold	Ours, 10-fold	Ours, 10-fold, nested-tuned	Published, LOSO	Ours, LOSO	LOSO, participant-normalised	LOSO, + 2 units of context
EEG	77.41	75.6	76.4	70.70	56.9	62.0	61.2
EEG+EDA	80.06	80.3	80.4	73.01	58.3	63.6	64.7
EEG+Gaze	80.75	80.0	81.0	74.68	59.8	63.0	63.3
EEG+EDA+Gaze	—	82.5	82.8	—	59.8	64.5	66.7
Clock (level, segment index)	—	71.5	—	—	73.7	—	—

Published: best accuracy reported by Angkan et al. for each modality combination (10-fold: random forest or XGBoost; LOSO: deep feature networks). Ours: untuned random forest of the main study. Nested-tuned: random

forest or extra trees, minimum leaf size 1 or 5 and feature fraction sqrt or 0.2, chosen by 3-fold cross-validation inside each outer training fold (code/run_nested_tuning.py). Participant-normalised: each feature z-scored over the participant's own segments (no labels). Context: mean over the current and two preceding units of the same drive. Gradient boosting was also tried in every LOSO configuration and reached 55.4% to 63.3%, against a best of 66.7% for the forest.

Table S20 CL-Drive: window position relative to the spoken report, position certificate and induced load (pooled ROC-AUC [participant-clustered 95% CI], leave-one-participant-out).

Window	Rating 5–9 vs 1–4, all	Rating increase (event), all	Rating increase (event), EEG
Report itself (+4.5 s)	0.582 [0.505, 0.674]	0.579 [0.510, 0.649]	0.567 [0.511, 0.629]
Report-free (+1.0 s)	0.593 [0.525, 0.672]	0.489 [0.425, 0.560]	0.505 [0.454, 0.556]
Report-free (−0.5 s)	0.593 [0.521, 0.676]	0.543 [0.479, 0.615]	0.519 [0.463, 0.586]
Report-free (−2.0 s)	0.576 [0.501, 0.661]	0.591 [0.527, 0.649]	0.578 [0.518, 0.645]
Previous report (−5.0 s)	0.559 [0.491, 0.641]	0.500 [0.451, 0.546]	0.497 [0.457, 0.541]
Published 10-s segment	0.560 [0.476, 0.650]	—	—

Event design: 93 reported increases, each with one unchanged-rating control before and one after it in the same drive, at least two units away. Steps on all features for the event design: +0.090, −0.054, −0.048; $L^* = 0$. Position certificate for the event design, on position: linear 0.547 [0.522, 0.568], spline 0.608 [0.554, 0.664], random forest 0.650 [0.568, 0.712], gradient boosting 0.643 [0.571, 0.702], 25-nearest neighbours 0.643 [0.580, 0.699]; on position and level: random forest 0.731 [0.651, 0.807], gradient boosting 0.728 [0.645, 0.801]. Clock for the rating target: 0.741 [0.693, 0.812]. Induced level, 7–9 against 1–3, report-free window: all sensors 0.926 [0.903, 0.947], EEG 0.809, EDA 0.812, gaze 0.859, segment index 0.500.
